# Editing of *SlWRKY29* by CRISPR-activation promotes somatic embryogenesis in *Solanum lycopersicum cv*. Micro-Tom

**DOI:** 10.1371/journal.pone.0301169

**Published:** 2024-04-01

**Authors:** Eliana Valencia-Lozano, José Luis Cabrera-Ponce, Aarón Barraza, Alberto Cristian López-Calleja, Elsa García-Vázquez, Diana Marcela Rivera-Toro, Stefan de Folter, Raúl Alvarez-Venegas

**Affiliations:** 1 Center for Research and Advanced Studies of the National Polytechnic Institute, CINVESTAV-IPN, Unidad Irapuato, Irapuato, Guanajuato, México; 2 Programa de Agricultura en Zonas Áridas, CONACYT-CIBNOR, Centro de Investigaciones Biológicas del Noroeste, La Paz, Baja California Sur, México; 3 Center for Research and Advanced Studies of the National Polytechnic Institute, CINVESTAV-IPN, Unidad de Genómica Avanzada, Irapuato, Guanajuato, México; Nuclear Science and Technology Research Institute, ISLAMIC REPUBLIC OF IRAN

## Abstract

At present, the development of plants with improved traits like superior quality, high yield, or stress resistance, are highly desirable in agriculture. Accelerated crop improvement, however, must capitalize on revolutionary new plant breeding technologies, like genetically modified and gene-edited crops, to heighten food crop traits. Genome editing still faces ineffective methods for the transformation and regeneration of different plant species and must surpass the genotype dependency of the transformation process. Tomato is considered an alternative plant model system to rice and Arabidopsis, and a model organism for fleshy-fruited plants. Furthermore, tomato cultivars like Micro-Tom are excellent models for tomato research due to its short life cycle, small size, and capacity to grow at high density. Therefore, we developed an indirect somatic embryo protocol from cotyledonary tomato explants and used this to generate epigenetically edited tomato plants for the *SlWRKY29* gene *via* CRISPR-activation (CRISPRa). We found that epigenetic reprogramming for *SlWRKY29* establishes a transcriptionally permissive chromatin state, as determined by an enrichment of the H3K4me3 mark. A whole transcriptome analysis of CRISPRa-edited pro-embryogenic masses and mature somatic embryos allowed us to characterize the mechanism driving somatic embryo induction in the edited tomato cv. Micro-Tom. Furthermore, we show that enhanced embryo induction and maturation are influenced by the transcriptional effector employed during CRISPRa, as well as by the medium composition and *in vitro* environmental conditions such as osmotic components, plant growth regulators, and light intensity.

## Introduction

As the world population continues to rapidly expand, it will be a significant and continuous challenge to improve and increase global food production. Meeting this challenge requires the development of innovative strategies for crop production and enhanced integrated pest management systems. Promising approaches include the development of new stress-resistant crop cultivars through conventional breeding, the incorporation of advantageous plant microbiomes into agricultural practices, and the development of genetically modified (based on the introduction of genes from another species by genetic recombination) and genome edited plants (which involves a direct use of DNA-cutting enzymes within cells and showing no exogenous genetic materials in its genome) [1,2]. The revolutionary CRISPR/Cas9 gene editing system, for instance, has emerged as a potent biotechnological tool facilitating targeted genomic alterations for studying gene function or enhancing valuable agronomic traits in crops [3–5].

Furthermore, a novel strategy for addressing emerging challenges in plant development is through epigenome editing. This technique involves the precise addition and/or removal of epigenetic marks to induce long-lasting changes in gene transcription. Many enzymes catalyzing epigenetic modifications regulate numerous developmental and physiological processes by directly influencing chromatin organization and gene transcription [6]. Consequently, epigenome editing involves the targeted modification of epigenetic marks at genomic loci using synthetic epigenome engineering tools [7,8]. Epigenome editing employs engineered nucleases, such as the nuclease-deficient Cas9 (dCas9), which retains its DNA-binding capability but is incapable of cleaving DNA [9–11]. Therefore, engineered nucleases such as dCas9 or dCas12a, can be fused to epigenetic regulatory factors to enable stable and efficient transcriptional repression or activation patterns (CRISPR interference or CRISPR activation, respectively) [12], without altering the DNA sequence.

In contrast to genetic editing, epigenetic editing is an emerging approach to enhance beneficial plant traits. For instance, CRISPR activation (CRISPRa)-mediated histone acetylation has been used to enhance drought stress tolerance in *Arabidopsis* [13]. Recently, we generated epigenetically edited tomato plants via CRISPRa, by fusing dCas9 to the catalytic SET-domain of the tomato *SlATX1* gene (ortholog to the histone H3 lysine 4 tri-methyltransferase *ATX1* gene from *Arabidopsis*) and showed that activation of defense genes protects tomato plants against pathogens [14]. Accordingly, up-regulation of target genes via synthetic epi-effectors is an attractive tool for targeted manipulation of the epigenome [7]. Therefore, epigenetic reprogramming or the ability to accomplish histone and DNA modifications will have a novel effect on plant breeding [15], and would be valuable both in plant genetic research and crop engineering. It is still essential, however, the regeneration of epigenetically edited plants through somatic embryogenesis (SEs). While SEs occurs in nature [16,17] and can also be induced *in vitro* under specific plant tissue culture conditions [18], achieving optimal plant conversion efficiency from somatic embryos remains a critical goal for developing new crop cultivars.

Tomato (*Solanum lycopersicum* L.) is the perfect crop for (epi)genome editing because of the information about its basic biology and genetics, sequenced genome, its economic importance, and efficient transformation methodology [19]. As most solanaceous species, tomato has been transformed and regenerated using indirect organogenesis, resembling a process that occurs in nature mainly by wounding [20]. Organogenesis, however, has been reported as incompatible for transgenic research due to the production of chimeric shoots (containing both transformed and untransformed cells) [21,22]. An alternative procedure to increase the efficiency of genetic transformation and (epi)genome editing, is by means of SEs, a process in which an embryonic stem cell is induced from a somatic cell that differentiates into a somatic embryo, with the capacity to develop a plant that contains the same genetic information as its precursor. Plant conversion rates, however, are still low and demand improvements to enhance the cost-effectiveness of commercial micropropagation [23].

WRKY proteins are plant-specific transcription factors involved in multiple biological processes, for instance, biotic and abiotic stress responses (heat, drought, salinity, and oxidative stresses) [24], secondary metabolism [25], and somatic embryogenesis [26]. Recently, we reported that in the common bean (*Phaseolus vulgaris*), enhanced resistance to *Pseudomonas syringae* pv. *phaseolicola* infection was conferred by the priming activator β-aminobutyric acid (BABA), and correlated with primed transcription of *PvWRKY6*, *PvWRKY29*, *PvWRKY53* [27]. In addition, transcriptome analysis has shown that expression of *WRKY* genes is induced in papaya [28] and *Arabidopsis thaliana* [29] embryogenic callus, supporting their possible significant role in the process of somatic embryogenesis. The WRKY transcription factor family is one of the largest in plants and a substantial number of *WRKY* genes are found in different species, for example, 74 in Arabidopsis, 197 in soybean, and 85 in tomato [24]. Hence, to enhance and optimize the SEs process and to surmount the inefficient plant regeneration approaches, we analyzed the relationship between CRISPRa-mediated transcriptional activation of the *SlWRKY29*, somatic embryogenesis, and somatic embryo-derived plant conversion.

The WRKY29 transcription factor has been linked to enhanced disease resistance, pattern-triggered immunity (PTI), and is also induced in response to, for example, *Fusarium graminearum* infection [30]. Furthermore, *WRKY29* regulates ethylene production in Arabidopsis [31], and is considered a marker gene of defense priming and plant immunity [32,33]. While its role in somatic embryogenesis has not been established, we hypothesized that the recruitment of epigenetic factors (e.g., histone lysine methyltransferases associated with activation of gene expression), via a defective Cas9 or Cas12a (dCas9 or dCas12a), can activate the expression of the *SlWRKY29* gene, which in turn enhances SE formation and germination. Thus, the goals of this work were to transform tomato explants, generate and select CRISPRa-edited tomato somatic embryos with enhanced *WRKY29* expression, and to analyze the transcriptome during the induction and germination of SE to identify potential genes involved in the process. To achieve this, we generated a set of binary vectors for epigenome editing in tomato via CRISPRa. Subsequently, cotyledonary explants from tomato Micro-Tom were transformed by biolistics and cultured with various combinations of plant hormones to induce CRISPRa-edited callus, embryo formation, embryogenic lines, and ultimately edited plants. Our findings indicate that the activation of *SlWRKY29* via CRISPRa upregulates a specific set of genes involved in SEs (e.g., *LEC1*, *FUS3*, *ABI5*, *WUS*, *WOX2*, *4*; as well as chromatin-remodeling proteins), and embryo germination and plant regeneration occurred faster and efficiently.

## Materials and methods

### Vector construction

For vector construction we have followed the protocols developed by Lowder and colleagues [34–36]. For a detailed description of vector construction see S1 Table. In summary, we generated a set of expression vectors (S1 Fig) to activate expression of the *SlWRKY29* gene via either a defective dCas9 (CRISPR-Act2.0 constructs) or dCas12 (CRISPR-dCas12 constructs) nucleases. dCas9 vectors (CRISPR-Act2.0 constructs) were designed to have a combination of two effector domains (SET-domain; separated by the bacteriophage coat protein MS2) [36], and were fused in tandem to its C-end (dCas9:SET-*MS2*-SET). dCas12 vectors consist of only one effector domain (SET-domain), fused at the C-end of the dCas12 nuclease (dCas12:SET). In addition, both series of expression vectors contain guide RNAs (gRNAs) to target the promoter region of the *SlWRKY29* (*Solanum lycopersicum WRKY29*; GeneID 101245784) and were designed with the help of the CRISPRP2.0 [37] and CHOPCHOP [38] DNA editing software. Three target sequences were selected for dCas9 (*SpCas9*, PAM: 5’-NGG-3’) and three for dCas12 (*LbCpf1*, PAM: 5’-TTTV-3’). S2 Table shows the different target sequences.

### Generation of cotyledon explants

Tomato Micro-Tom seeds (Moles Seeds, cat. # VTO325) were surface sterilized, rinsed and soaked for 24 h in smoke-water (0.5% v/v) [39], and then rinsed for 1 min in three changes of sterile distilled water. Next, seeds were manually scarified, and then placed on MS basal medium (Sigma-Aldrich cat. # M5519) [40] supplemented with 3g/L activated charcoal plus 3 g/L Gelrite (Sigma-Aldrich, Gelzan CM, Gelrite cat. # G1910) and incubated in a Percival growth chamber (Percival AR-36, Percival, Perry, IA, USA) at 22°C under long day conditions (16 h light/8 h darkness, with an irradiance of 50 μmol/m^−2^ s^−1^, using fluorescent T8 Phillips P32T8 /TL850 lamps). After 8 days, the seedlings were dissected using a stereo microscope as follows: the cotyledons were removed from the embryonic axis using a dissecting scalpel and 3 mm long cotyledon explants were used for transformation.

### Microprojectile bombardment and plant regeneration protocols

Before being subjected to particle bombardment, cotyledon explants were sub-cultured for 24 h in three different osmotic treatment media: MS-BK2iP, containing 5% sucrose and 5 g/L gelrite (described here as high osmotic medium; modified from [41–43]); MS-NAA/BAP, containing 3% sucrose (considered here as a conventional osmotic medium, modified from [44]); or MS-Zea media, containing 5% sucrose and 5 g/L gelrite (described here as high osmotic medium; modified from [41–43] (see S4 Table for medium components). Each experimental unit (bombarded plate) contained 20 explants of 3 mm^2^, and 9 plates for each CRISPRa construction were used (S2 Fig). A Bio-Rad PDS-1000/He particle delivery system was used to bombard, following established protocols [45], the cotyledon explants (abaxial side upwards) that were kept on the different treatment media. Control samples were bombarded with the control empty vectors or without DNA (see S1 Fig and S3 Table for a list of the different constructs used).

### Selection of CRISPRa-edited plants

After bombardment, all explants were kept for twenty-four hours in the same media described before, and then sub-cultured on conventional osmotic medium (MS-BK2iP, MS-BAP, or MS-Z; S4 Table), without antibiotics. After 8 days, explants were transferred to selective medium (MS-BK2iP, MS-BAP, or MS-Z, correspondingly), containing hygromycin 9.5 mg/L (see S4 Table), and incubated for two weeks in a growth chamber at 22°C under 16 h light/8 h darkness, with an irradiance 50 μmol/m^−2^ s^−1^ (fluorescent T8 Phillips P32T8 / TL850 lamps). Four rounds of two weeks incubation were applied. Subsequently, putative CRISPRa-edited embryogenic structures (somatic embryos) were dissected and individually sub-cultured for 15 days onto fresh selective medium for pro-embryogenic mass formation. Then, pro-embryogenic masses (embryogenic lines, or PEMs) were regularly sub-cultured (at 2-week intervals, 3–4 times) onto fresh selective medium until secondary embryogenesis was observed (S2A–S2F Fig).

For all embryogenic lines, genomic DNA was extracted from PEMs with the Plant DNAzol Reagent (Invitrogen, cat. #10978021; following the manufacturer’s instructions), and the presence of the transgene was confirmed by PCR with 35SCaMV, dCas9 and dCas12 specific primers (S2G Fig; see S2 Table for a list of the primers used). Only edited positive embryogenic lines (growing on selective medium), were chosen for further experiments.

Once the embryogenic lines were established, and to study the effect of exogenous application of cytokinin on the activation of the shoot apical meristem (SAM) and root apical meristem (RAM) during somatic embryo maturation, two different treatments were evaluated (G9-2iP or G9-Zea maturation, rooting and elongation media; S4 Table). Plates containing individualized somatic embryos were incubated at 25 ± 2°C, under a 12/12 h photoperiod at 50 μmol/m−2 s−1 irradiance provided by fluorescent lamps T8 Phillips P32T8/TL850. After observing elongation of the embryos (30 days, approximately), they were individually sub-cultured for 30 days, on the same media, until the RAM was formed. Then, embryos were transferred to clear plant tissue culture glass bottles (for rooting and elongation) and kept for 60 days in a growth chamber at 22°C under long-day light cycles (as mentioned before). Next, whole individual plants, 5 to 15 cm in length, were transferred to 4.3 L plastic pots (Sunshine Mix #3 potting mix, Sun Grow Horticulture, USA) and placed in the greenhouse (14 h photoperiod, with an average temperature of 18–25°C).

### Histological analysis

Randomly chosen masses from embryogenic lines, as well as secondary somatic embryos, were collected and fixed in 1 mL of FAE (3.7 % Formaldehyde, 10 % Acetic acid glacial, 50 % Ethanol) for two hours, at room temperature, followed by dehydration in a series of ethanol dilutions, as described by [14]. Fixed and dehydrated samples were embedded in Technovit 7100 (Heraeus Kulzer, cat. #64709003) plus ethanol (1:1, v/v), and then embedded in the infiltration solution (Technovit 7100 plus hardener), according to the manufacturer´s instructions. Sections of 10-μm were prepared using a microtome as previously described [14]. Pictures were taken and analyzed on an upright Leica DM6000B microscope.

### Isolation of RNA and gene expression analysis

Total RNA from PEMs (cultured in MS-BK2iP medium) and individual embryos (15-days after being cultured in G9-2iP media), was isolated using Trizol (Invitrogen, Carlsbad, CA, USA). RT-qPCR was performed as previously described [14]. A Step-One® Real-time PCR system (Applied Biosystems, Foster City, CA, USA) was used for real-time PCR quantifications. Fold change of the *SlWRKY29* gene was normalized to the reference genes *SlLSM7* and *SlTIP41* [46,47]. Relative expression of the *FIE*, *WUS*, *LEC1*, and *FUS3* tomato gene homologs was normalized to the *SlLSM7* reference gene. Data from qPCR experiments were analyzed based on the 2^−ΔΔCT^ method [48]. qPCR analysis was based on three biological replicates for each sample, with three technical replicates. For a list of all primers used see S2 Table.

### Chromatin immunoprecipitation

Chromatin isolation and immunoprecipitation were performed as previously described [14], with some modifications. In brief, 500 mg of PEMs or 15-d embryos were used for chromatin isolation. The chromatin was digested to ~200 bp fragments with 20 U of micrococcal nuclease (Catalog #88216, Thermo Scientific) at 37°C for 30 min. Each ChIP experiment was independently performed in duplicate. PCR amplification was carried out by using the Maxima SYBR Green/ROX qPCR Master Mix (Catalog # K0222). Primers used were as follows: F 5’-tggggtcttcaagctgttgtt-3’; R 5’-ccaccatcaacatgataaaatggct-3’ (primer set amplifies region +16–206 nt of the *SlWRKY29* gene). All PCR reactions were run in triplicate (technical replicates per sample). ChIP-qPCR data was normalized versus the input sample (1% starting chromatin), according to the Percent Input method *(% Input*  =  2^((Cq(IN)-Log^_2_^(DF))-Cq(IP))^ * 100) [49].

### RNA-seq and differential gene expression analysis

Similarly, for RNA extraction and subsequent transcriptome sequencing, 100 mg tissue from PEMs (cultured in MS-BK2iP medium) and individual embryos (15-days after being cultured in G9-2iP media), of the FS1H and CT2H samples (from three independent transformation events or embryogenic lines each), were processed to obtain total RNA (Trizol reagent; Invitrogen, Carlsbad, CA, USA). The RNA was quantified on a Nanodrop 2000 (Thermo Fischer Scientific), and the quality was verified on a 2% agarose gel. Azenta Life Sciences RNA Sequencing Services for RNA-seq sample preparation and sequencing were used (https://www.genewiz.com//en/Public/Services/Next-Generation-Sequencing/RNA-Seq). Briefly, total RNA integrity was assessed on an Agilent 4200 TapeStation with RNA ScreenTape & Reagents (Agilent). Samples with a RIN number ≥ 6.0 were enriched, followed by library preparation (S5 Table shows the barcode sequences used), using the Illumina TruSeq Stranded Total RNA kit (following the manufacturer’s protocol). A total of 18 paired-end libraries were sequenced, via an Illumina^®^ HiSeq^TM^ system (2x150 bp configuration), to generate ~350M raw paired-end reads per lane (~30-40M reads per sample). Two technical sequencing replicates for each biological sample were conducted. Sequence reads were trimmed to remove adapter sequences and nucleotides with poor quality using Trimmomatic v.0.36. The trimmed reads were mapped to the Tomato reference genome available on ENSEMBL using the STAR aligner v.2.5.2b. BAM files were generated as a result of this step. Unique gene hit counts were calculated by using featureCounts from the Subread package v.1.5.2. The hit counts were summarized and reported using the gene_id feature in the annotation file. Only unique reads that fell within exon regions were counted. After extraction of gene hit counts, the gene hit counts table was used for downstream differential expression analysis. Using DESeq2, a comparison of gene expression between the defined groups of samples was performed [50]. The original values were normalized and used to accurately determine differentially expressed genes. The Wald test was used to generate p-values and log2 fold changes. Genes with an adjusted p-value < 0.05 and absolute log2 fold change > 1 were called as differentially expressed genes for each comparison. Also, the original values were normalized to adjust for various factors such as variations in sequencing amount. These normalized values were used to accurately determine differentially expressed genes. The datasets and accession number(s) presented in this study can be found online at NCBI, https://www.ncbi.nlm.nih.gov/bioproject/PRJNA915758.

## Results

### Induction of somatic embryogenesis and development of embryogenic lines

Before transformation, cotyledonary explants from *S*. *lycopersicum* cv. Micro-Tom were cultured for 24 h in different stress treatment media: two different high osmotic mediums containing distinctive plant hormones from the cytokinin family (trans-Zeatin; N6-(Δ2-isopentenyl)adenine, 2iP; and 6-Benzylaminopurine, BAP), and one conventional osmotic medium containing a synthetic auxin (naphthaleneacetic acid, NAA) (see S4 Table). Then, explants were transformed by biolistics with the different CRISPRa constructs (S1 Fig), kept for another 24 h after transformation in the same osmotic treatment media, and then sub-cultured to non-osmotic conventional medium without antibiotics (S4 Table), for 8 days.

Next, explants were transferred to selective medium containing hygromycin and, after two months, PEMs developed differentially on each selective medium evaluated (some explants were necrotic and died, while others developed green PEMs; S2B and S2C Fig). Accordingly, SEs was induced from cotyledonary explants since nutrients easily penetrate such tissue and consequently facilitates antibiotic selection and the effective suppression of non-transformed cells [51] Furthermore, we detected that on the MS-BK2iP selective medium there were a higher number of explants producing PEMs (11.1 to 33.3% efficiency), and a higher number of PEMs producing somatic embryos (60 to 100% efficiency), when compared to the other two mediums (MS-NAA/BAP and MS-ZEA) (see Table 1). Particularly, FSH1 (dCas12:SET+gRNA)-transformed explants, sub-cultured on MS-BK2iP selective medium, gave the highest number of explants producing PEMs (60 explants producing PEMs, from a total of 180 explants), and the highest number of PEMs producing somatic embryos (60/60 PEMs producing SE) (Table 1A), when compared to TR2H (dCas9:SET-*MS2*-SET+gRNA) and control samples.

**Table 1:** Efficiency of explants producing PEMs, and PEMs producing somatic embryos, for all edited lines on the different selective mediums. **A)** MS-BK2iP. MS basal medium supplemented with 1 mg/L 2iP (N6-(Δ2-isopentenyladenine), 1 mg/L kinetin, 2 mg/L BAP, and 9.5 mg/L of hygromycin. **B)** MS-NAA/BAP. MS basal medium supplemented with 1 mg/L of BAP and 9.5 mg/L hygromycin. **C)** MS-ZEA. MS basal medium supplemented with 1.5 mg/L trans-Zeatin, and 9.5 mg/L of hygromycin.

Editing plasmid	Detableription	# of explants producing PEMs - Efficiency %	# of PEMs producing SE - Efficiency %
CT2H	Hyg:GFP	20/180 = 11.1	12/20 = 60
FS1H	dCas12:SET+ gRNA	60/180 = 33.3	60/60 = 100
SL0H	dCas12:SET	50/180 = 27.8	44/50 = 88
TR2H	dCas9:SET-MS2-SET+gRNA	47/180 = 26.1	46/47 = 98
RZ0H	dCas9:SET-*MS2*-SET	39/180 = 21.7	33/39 = 85
CT2H	Hyg:GFP	8/180 = 4.4	4/8 = 50
FS1H	dCas12:SET+ gRNA	34/180 = 18.9	34/34 = 100
SL0H	dCas12:SET	29/180 = 16.1	25/29 = 86.2
TR2H	dCas9:SET-MS2-SET+gRNA	28/180 = 15.6	27/28 = 96.4
RZ0H	dCas9:SET-*MS2*-SET	22/180 = 12.2	19/22 = 86.4
CT2H	Hyg:GFP	15/180 = 8.3	8/15 = 53.3
FS1H	dCas12:SET+ gRNA	50/180 = 27.8	49/50 = 98
SL0H	dCas12:SET	41/180 = 22.8	39/41 = 95.1
TR2H	dCas9:SET-MS2-SET+gRNA	39/180 = 21.7	38/39 = 97.4
RZ0H	dCas9:SET-*MS2*-SET	33/180 = 18.3	30/33 = 90.9

**Fig 1:**
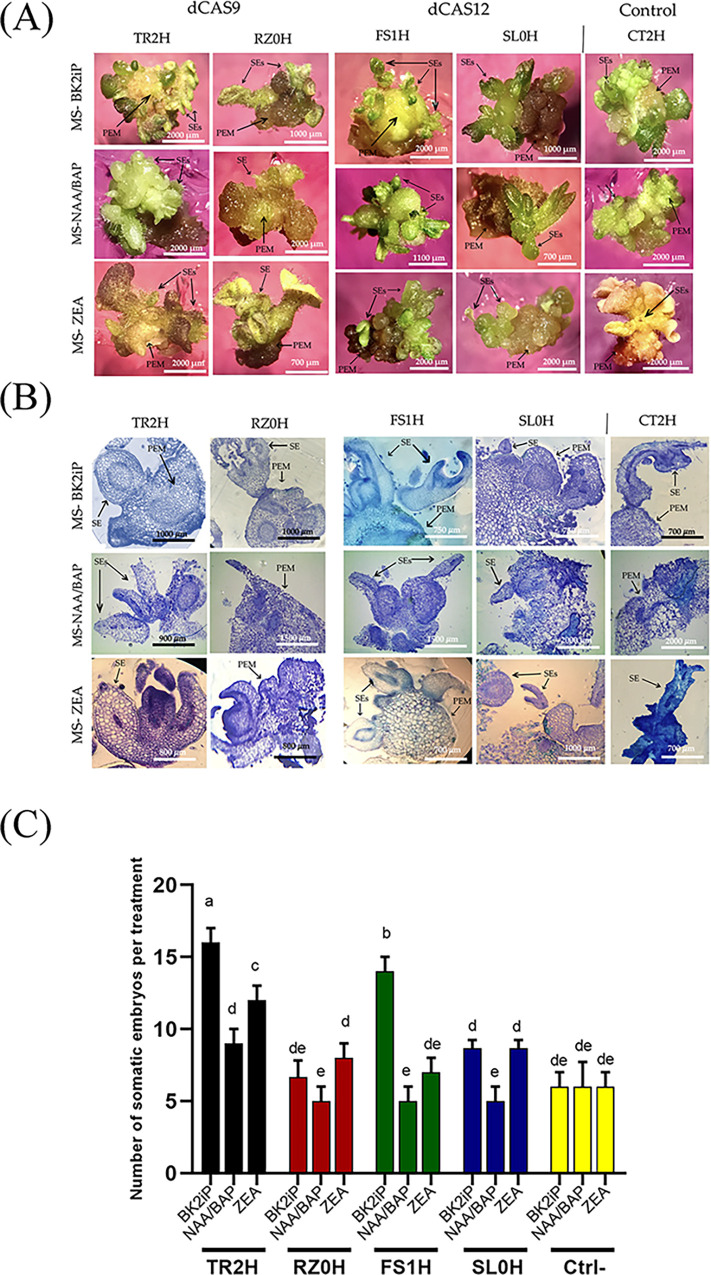
Induction of pro-embryogenic masses and secondary embryogenesis in three different hygromycin containing culture media. (A) Individual transformation events or somatic embryos, transformed with the different CRISPR-Act2.0 dCas9 or CRISPR-dCas12 expression vectors, were sub-cultured onto fresh selective medium (MS-BK2iP, MS-NAA/BAP or MS-ZEA, as indicated), to evaluate the development of embryogenic structures on tomato pro-embryogenic masses (PEMs). (B) Histological confirmation of somatic embryogenesis on tomato PEMs. (C) Number of secondary somatic embryos per PEM in three different selective media (BK2iP, NAA/BAP, ZEA). Average number of individual somatic embryos correspond to 20 independent transformation events, or lines, per construct. Letters represent the level of statistical significance (LSD-test) as determined by a two-way ANOVA (p-value <0.001). Means with the same letter are not significantly different.

After three to four rounds of incubation in selective medium, individual edited embryogenic structures per PEM (and for each plasmid construction) were dissected and separately sub-cultured onto fresh selective medium. Subsequently, after PEMs formed, the presence of the transgene was confirmed by PCR (S2 Fig) and PEMs were regularly sub-cultured onto fresh selective medium until secondary embryogenesis was observed (Fig 1A). Histological analysis showed evidence of SEs on PEMs. Somatic embryos (SE) were distinguished by a clear-cut uniform protoderm consisting of rectangular cells. In addition, heart and late cotyledonary-stage somatic embryos were loosely attached to the epidermal cells of the mother tissue and were regularly detached during sectioning (Fig 1B).

After evaluating the number of secondary SE formation, we concluded that the highest number of SE developed on the MS-BK2iP selective medium, followed by MS-ZEA (Fig 1C). Particularly, the number of individual somatic embryos obtained per PEM was dependent on the type of vector (treatment) used. As shown in Fig 1C, the number of somatic embryos produced in the TR2H line (dCas9:SET-*MS2*-SET+gRNA), when grown on the MS-BK2iP medium, corresponded to 16 SE per PEM; followed by the FS1H line (dCas12:SET+gRNA), with 14 SE per PEM; and then the control line SL0H (dCas12:SET) with 8 and RZ0H (dCas9:SET-*MS2*-SET) with 6 SE per PEM (compared to the 6 SE per PEM for the CT2H empty vector). In contrast, the number of SE produced per PEM, when grown on the MS-ZEA medium, corresponded to 12 SE per PEM for the TR2H line (dCas9:SET-*MS2*-SET+gRNA), followed by SL0H line with 9 and RZ0H line with 8 SE per PEM, and at the end, the FS1H line with 7 SE per PEM (compared to the 6 SE per PEM for the CT2H empty vector).

These results suggest an improved SE production, via CRISPRa-mediated transcriptional activation of the *SlWRKY29* gene, when using the MS-BK2iP medium before and after transformation (from high sucrose concentration during osmotic stress to low or conventional sucrose concentration during selection, respectively). Accordingly, we selected the MS-BK2iP as the principal selective medium for further experiments.

### Regeneration of edited plants

**Fig 2:**
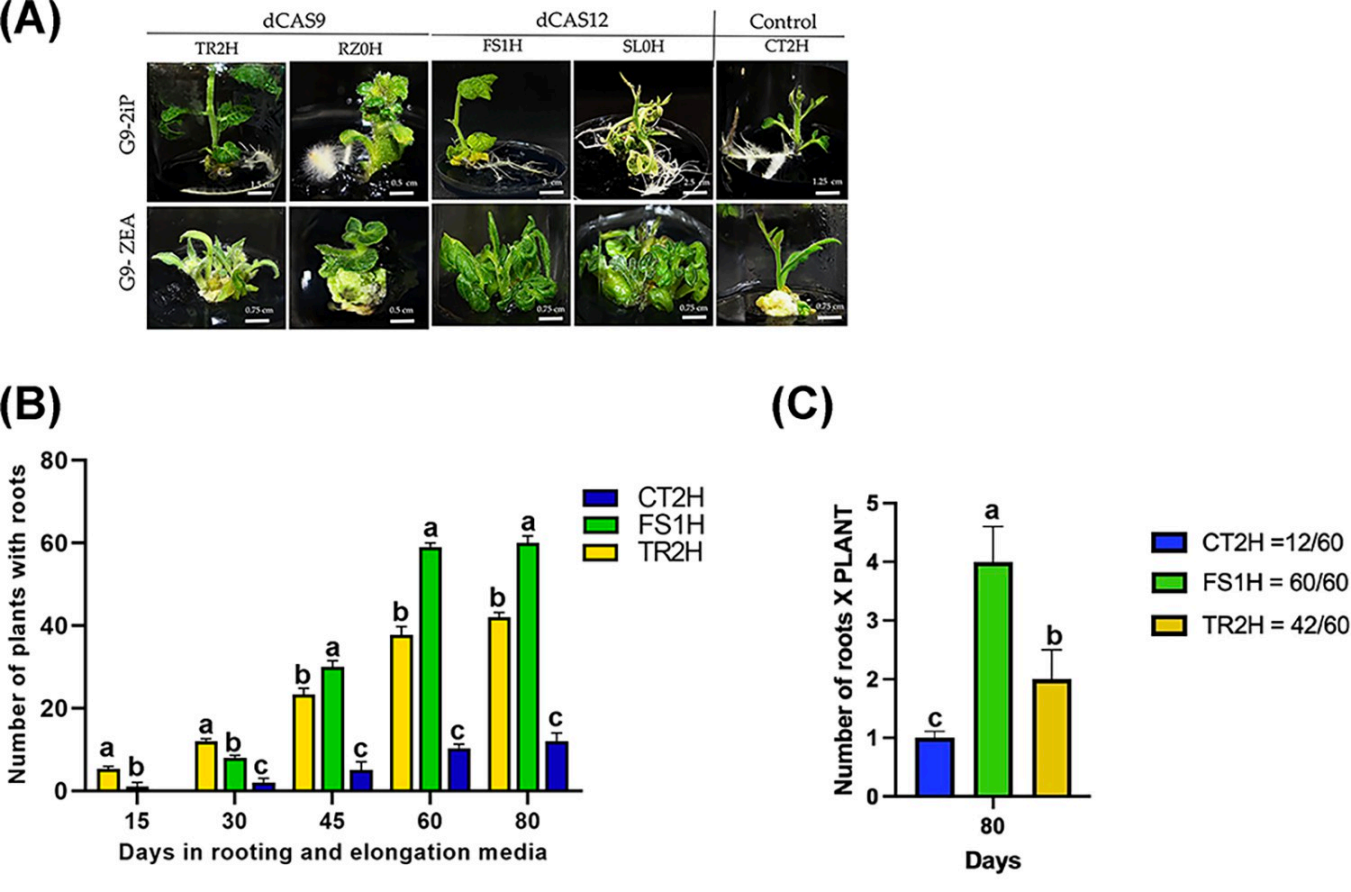
Plant development on maturation, rooting and elongation medium. (A) Individual embryos, taken from the different embryogenic lines, were transferred to G9-2iP or G9-Zea maturation, rooting and elongation medium and, after 30 days, they were sub-cultured into clear plant tissue culture glass jars containing fresh medium, for 60 more days. (B) Embryo maturation and root formation on G9-2iP maturation, rooting and elongation media. Sixty individual embryos from each of the TR2H, FS1H and CT2H lines were transferred to the G9-2iP medium and the number of plantlets with roots (additive measure) was registered at the indicated time points. At day 80, 100% (60/60) of FS1H plants developed roots, whereas 70% (42/60) and 20% (12/60) of TR2H and CT2H plants, respectively, developed roots. Statistical significance, at each timepoint, was determined using one-way ANOVA followed by Tukey’s test, p < 0.001. Means with the same letter are not significantly different. (C) Average number of roots per plant, after 80 days on G9-2iP medium, for each of the TR2H, FS1H and CT2H lines. Statistical significance was determined using one-way ANOVA followed by Tukey’s test, p < 0.001.

Once the embryogenic lines were established, and to assess the effect of exogenous application of cytokinin in the activation of the SAM and RAM during somatic embryo maturation, two different media for embryo maturation, rooting and elongation were evaluated: G9-2iP medium and G9-Zea medium (see S4 Table for medium components). Plates containing somatic embryos were incubated at 25 ± 2°C, under a 12/12 h photoperiod at 50 μmol/m−2 s−1 irradiance. After 30 days they were sub-cultured into clear plant tissue culture glass jar with plastic cap, containing fresh medium. After 60 days, plantlets developed in both types of medium and for all lines (Fig 2A). However, on G9-Zea medium strikingly the shoots did not developed roots, whereas on G9-2iP medium a typical shoot and root development was observed. This could indicate that Zea and 2-iP induced different auxin biosynthetic pathways. Table 2 shows some of the plants’ phenotypic characteristics after 90 days of embryo to plantlet conversion.

**Table 2:** Plant development in G9-2iP or G9-Zea maturation, rooting and elongation medium after 90 days of embryo to plantlet conversion.

Treatment	G9-2iP	G9-2iP	G9-2iP	G9-Zea	G9-Zea	G9-ZEA
	Plantlet height	Root length	Observations	Plantlet height	Root length	Observations
	(in cm ±SD; n = 20 plants)	(in cm ±SD; n = 20 plants)		(in cm ±SD; n = 20 plants)	(in cm ±SD; n = 20 plants)	
TR2H	7.1±2.3	9.3±1.5	Normal and simultaneous development of SAM and RAM; large leaflets; pubescent root	2.7±1.1	0±0	Leaf elongation; presence of several stems; without root development; callus-like structure
RZ0H	2.5±0.3	1.5±0.3	Rosette-like development; small leaflets; short stem	2.1±0.6	0±0	Rosette-like development; without root formation (callus-like structure)
FS1H	7.3±1.1	8.1±1.3	Large leaflets; fast development of stem and leaves; large number of secondary roots (6±2)	4.3±1.3	0±0	Leaf and stem elongation; vitrified (hyperhydrated); without roots
SL0H	5.6±0.7	7.2±0.6	Fast development of stem and leaves; large number of secondary roots (9±1.64)	2.5±1.0	0±0	Leaf and stem elongation; vitrified (hyperhydrated); without roots
CT2H	1.8±1.9	4.1±3.3	Slow development of stem and leaflets; pubescent root	3.1±1.3	0±0	Stem elongation, small leaflets; without roots; callus-like structure

Additionally, sixty individual embryos from each of the TR2H, FS1H and CT2H lines were transferred to G9-2iP medium and the number of plantlets with roots was determined. As shown in Fig 2B, TR2H plants on G9-2iP medium began developing roots earlier than FS1H or CT2H plants. However, after 80 days in rooting and elongation medium, 100% (60/60) of FS1H plants developed roots, whereas 70% (42/60) and 20% (12/60) of TR2H and CT2H plants, respectively, developed roots. Furthermore, 45% of TR2H plants had on average two roots (2 to 3 cm long; Fig 2C); whereas FS1H plants which began developing roots later than TR2H plants, were vigorous in size and with more roots (four roots on average, 5 to 6 cm long; Fig 2C). In contrast, after 80 days in rooting medium, CT2H control plants had on average one root (1 to 2 cm long), and with a tendency to form organogenic-like aggregates in the remaining sub-cultured embryos. Next, individual plants were transferred to plastic pots and placed in the greenhouse.

### Histological observations

To corroborate the morphological differentiation and structure formation of somatic embryos, further histological analyses were performed. We randomly selected PEMs (~1.0 cm in diameter) from three independent transformation events or embryogenic lines (developed from individually sub-cultured somatic embryo-like structures), growing on the key selective medium (MS-BK2iP), and for the two constructs producing the highest number of embryos (TR2H and FS1H; see Fig 1C). In addition, after 7 and 15 days, individual embryos sub-cultivated in G9-2iP (maturation, rooting and elongation medium) were also analyzed. CT2H samples, containing the empty vector, were used as control.

**Fig 3:**
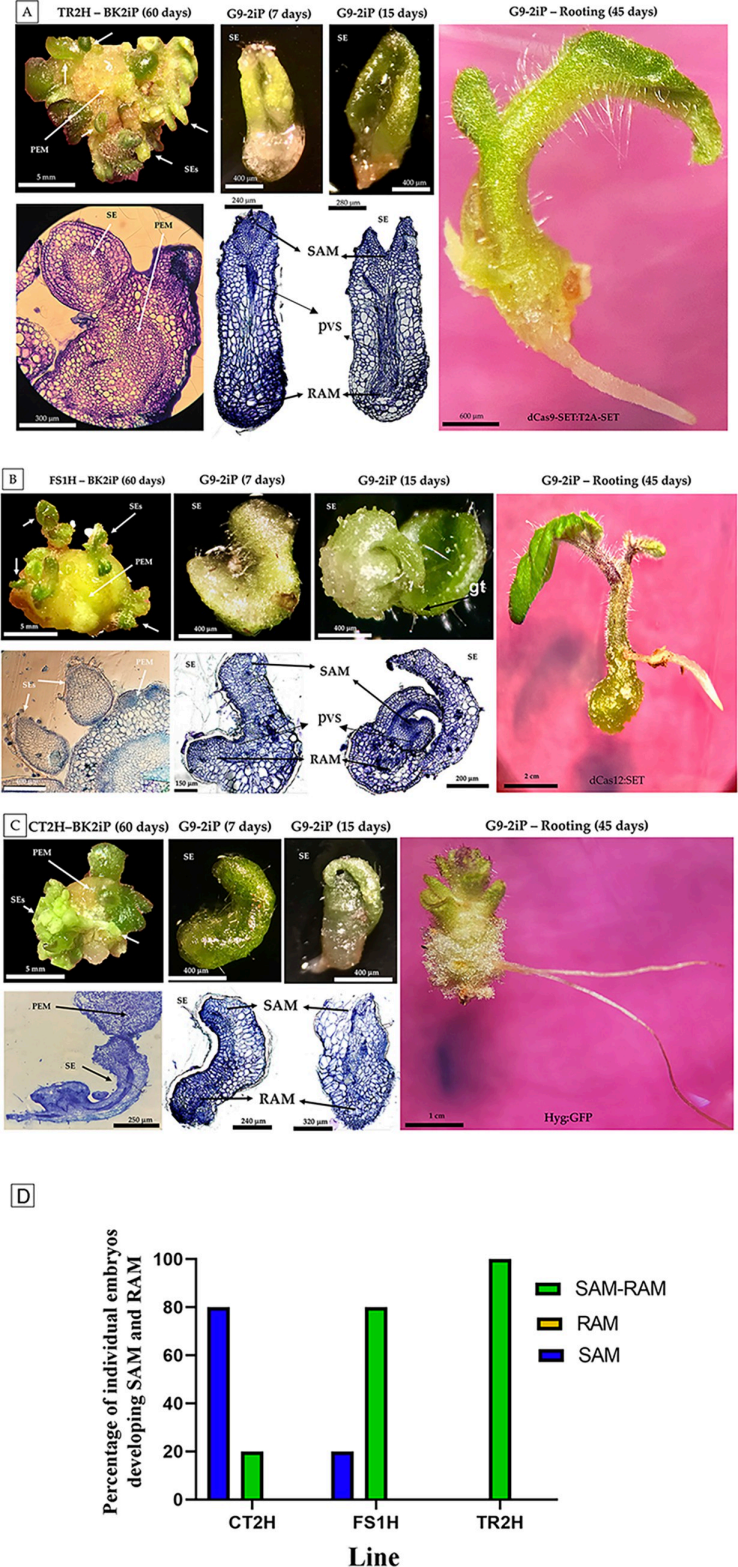
Histological evidence of somatic embryogenesis in tomato. (A) TR2H. (B) FS1H. (C) CT2H. A-C shows light micrographs of PEMs growing on BK2iP medium and SE after transfer to G9-2iP medium; cross-sections of PEM (60 days-old embryogenic lines), SE during secondary embryogenesis (7 and 15 days after transfer to G9-2iP medium); and plantlets (45 days after transfer to G9-2iP medium). (D) Percentage of individual somatic embryos developing SAM and RAM when grown in G9-2iP medium (n = 15 individual somatic embryos). Abbreviations: SE, somatic embryo; PEM, pro-embryogenic mass; SAM, shoot apical meristem; RAM, root apical meristem; pvs, provascular system; gt, glandular trichome.

As shown in Fig 3, SE differentiated from PEMs in all treatments. The development of a vascular axis was present seven days after sub-culture. Moreover, fifteen days after sub-culture, TR2H and FS1H individuals showed a typical SAM and RAM (bipolar structures with an apical and a basal pole; Fig 3A and 3B). Furthermore, from five individual somatic embryos per embryogenic line, grown on G9-2iP medium and analyzed under the microscope, a 100% of TR2H embryos developed shoot and root apical meristems; 80% of FS1H samples developed shoot and root apical meristems; whereas in the CT2H control sample only 20% of SE presented both SAM and RAM, while the other 80% only developed SAM (Fig 3D). Additionally, individuals from the FS1H treatment also developed glandular trichomes on the surface area of each individual somatic embryo (structures identified as defense mechanisms in different crops; Fig 3B) [31].

### *SlWRKY29* expression and ChIP assay in CRISPRa-activated PEMs and embryos

**Fig 4:**
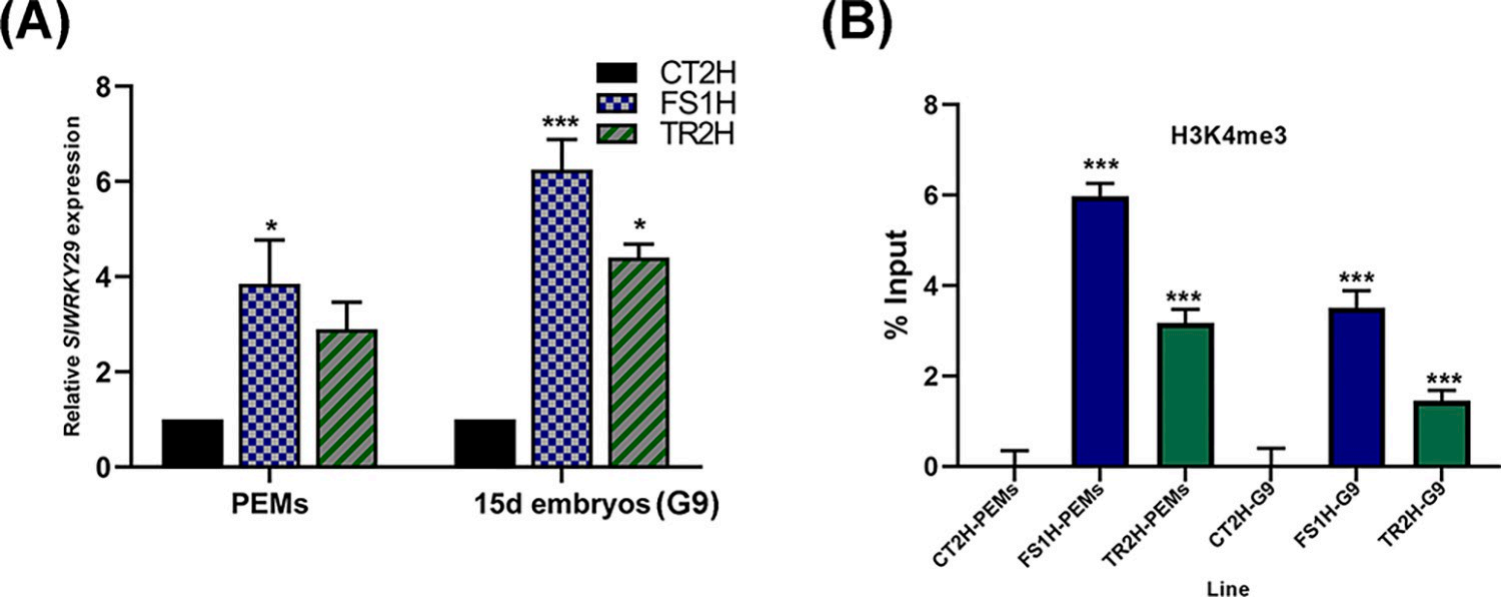
*SlWRKY29* gene expression and ChIP assay. (A) Transcript levels of *SlWRKY29* in edited PEMs and 15-days embryos. Samples were taken from pro-embryogenic masses (PEMs) and 15-days somatic embryos (in G9-2iP), and the relative expression of *SlWRKY29* was determined by qPCR reactions. Data were normalized independently to the *SlLSM7* and *SlTIP41* reference genes (based on the 2^−ΔΔCT^ method) [48], and results are shown as a combined set. Data represent mean ± SD from three independent experiments (n  =  3). (B) Chromatin immunoprecipitation assays to determine the presence/absence of the histone H3 lysine-4 trimethylation mark (H3K4me3) at the 5’-end region of *SlWRKY29* gene. ChIP assays were determined from pro-embryogenic masses (PEMs) and 15-days somatic embryos (G9). Data are mean ± SD. Each ChIP experiment was independently performed in duplicate (technical replicates, per line) from two biological replicates. ChIP-qPCR data was normalized versus the input sample (1 % starting chromatin), according to the Percent Input method *(% Input*  =  2^((Cq(IN)-Log^_2_^(DF))-Cq(IP))^ * 100) [49]. Statistical significance was determined with an unpaired two-tailed Student’s t-test (*p < 0.05, *** p < 0.001).

We determined the expression level of *SlWRKY29* by reverse transcription-quantitative PCR (RT-qPCR). Compared to CT2H control sample (as shown in Fig 4A), transcript levels of *SlWRKY29* in PEMs and elongated embryos (15 days in G9-2iP medium) were higher in both FS1H (3.8- and 6.0-fold, respectively) and TR2H (3.0- and 4.4-fold, respectively) samples, indicative of CRISPRa-mediated transcriptional activation of the *SlWRKY29* gene in edited PEMs and embryos. Furthermore, *SlWRKY29* expression in the FS1H line was higher in both PEMs and elongated embryos, when compared to PEMs and embryos of the TR2H line (once normalized with the *SlLSM7* and *SlTIP41* endogenous genes).

Next, we asked if the increased *SlWRKY29* transcript accumulation was associated with changes in the chromatin structure at the 5′-end region of *SlWRKY29*. Accordingly, we performed ChIP assays, using an antibody against the H3K4me3 mark, to gain insight into the status of the +1 nucleosome region, which we hypothesized is modified by the recombinant nucleases, dCas12:SET (FS1H vector) or dCas9:SET-*MS2*-SET (TR2H vector), which are guided to the 5′-end region by the specific gRNAs. ChIP analysis of the H3K4me3 mark showed that there is a 5.98-fold increase in H3K4me3 bound to the 5′-end chromatin region of *SlWRKY29* in FS1H PEMs, and a 3.17-fold increase in TR2H PEMs (when compared to the control CT2H-PEMs) (Fig 4B). In contrast, 15-days old individual embryos sub-cultivated in G9-2iP showed a 3.15- and a 1.45-fold increase in H3K4me3 bound to the 5′-end chromatin regions of *SlWRKY29* for the FS1H and TR2H lines, respectively (when compared to control CT2H-15d embryos). Interestingly, increase in the H3K4me3 mark correlates with the *SlWRKY29* expression levels in the FS1H and TR2H lines, for both PEMs and elongated embryos (15 days in G9-2iP medium).

Afterwards, to get a better understanding on the induction of somatic embryogenesis and somatic embryo-derived plant conversion, in CRISPRa-edited individuals, we selected for further experiments an edited line producing a high number of embryos, a greater number of plantlets with roots, and holding the greatest *SlWRKY29* expression, as well as the best medium that favors embryo germination and plant rooting and elongation (G9-2iP medium). Accordingly, samples from the FS1H embryogenic line (PEMs) before secondary SE induction and under embryogenic conditions (15 days in G9-2iP medium) were collected for RNA-Seq sequencing.

### Transcriptome analysis of differentially expressed genes during the transition from pro-embryogenic masses to embryo maturation at germination stage in *SlWRKY29* CRISPRa-edited tomato plants

The induction of SEs and embryo maturation are accompanied by complex mechanisms such as internal and external stimuli recognition, as well as by switching on and off gene regulatory networks. Therefore, an overall RNA-Seq screening was implemented for transcriptome analysis to get a broad view of possible regulatory networks and pathways involved in SEs and to identify potential genes involved in the induction and germination of *SlWRKY29* CRISPRa-edited SE.

As mentioned, we determined that a combination of cytokinins (kinetin, 2iP and BAP), was the best condition (after explant transformation) for PEM production, whereas for embryo maturation and plant development the best medium included kinetin and 2iP. Accordingly, the optimal sequential medium treatments for plant regeneration were as follows: MS-BK2iP containing 5% sucrose and 5 g/L gelrite (described here as high osmotic medium), 24 h before and after transformation; MS-BK2iP non-osmotic medium (conventional medium) for recovery after transformation; MS-BK2iP containing hygromycin as selective medium; and G9-2iP medium for embryo maturation, rooting and elongation (see S4 Table for media components).

**Fig 5:**
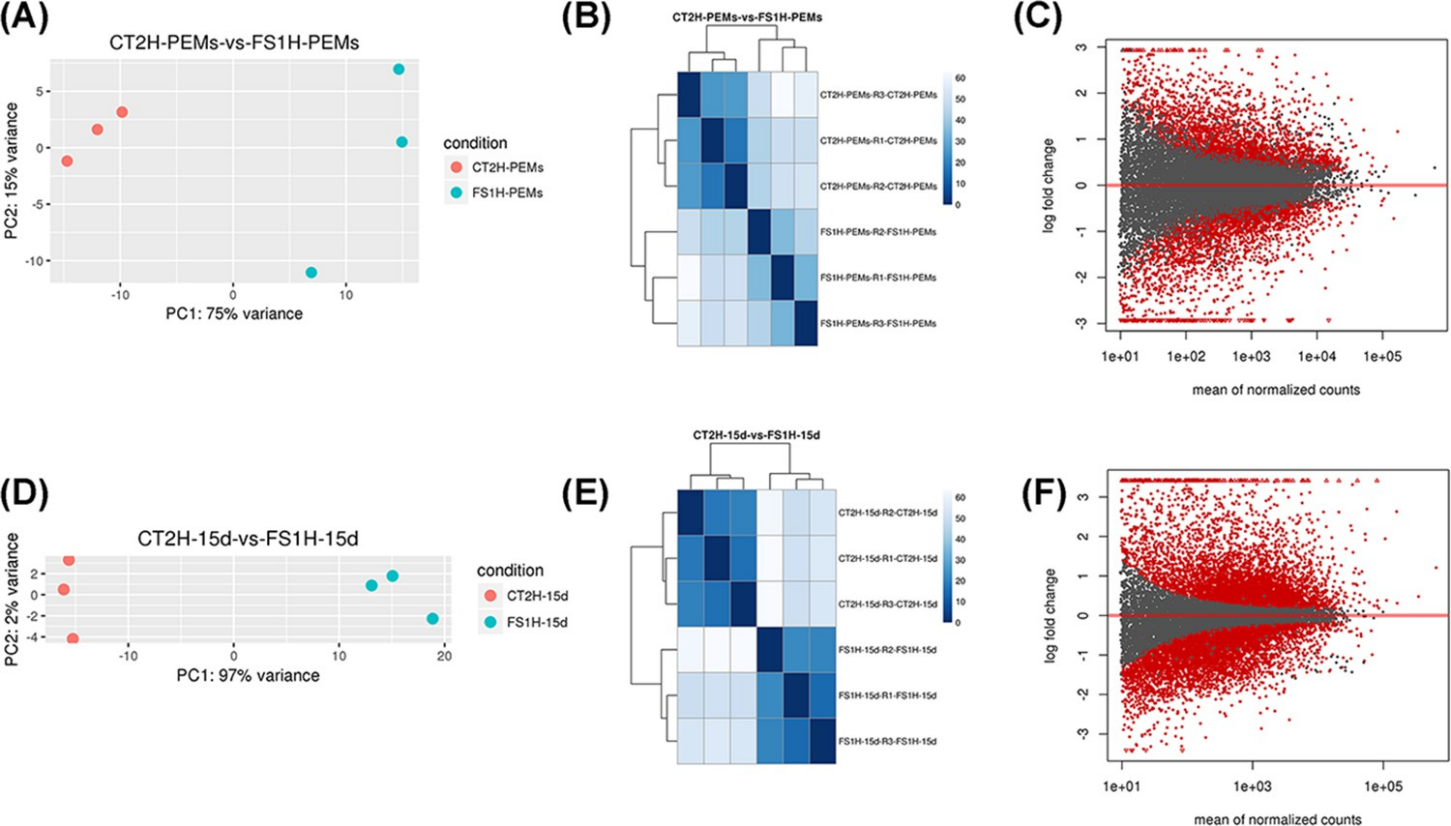
Dispersion analysis between samples. RNA-seq was applied to identify potential genes involved in the induction and germination of CRISPRa-edited SE. (A, D) Principal component analysis (PCA). PCA revealed the variance among each group. (B, E) Hierarchical biclustering analysis. Correlation heatmap results were consistent with PCA and showed homogeneity within the groups. (C, F) Mean-difference plots showing the log-fold change and average abundance of the differentially expressed genes (significantly up and down DEGs are highlighted in red). Data correspond to CT2H and FS1H samples at PEMs (A-C) and 15 days-embryos (D-F) stages, respectively.

Consequently, PEM samples growing on MS-BK2iP selective medium, before somatic embryo induction, and under embryogenic conditions (secondary SE on G9-2iP maturation medium, after 15 days), were collected for transcriptome analysis from FS1H (dCas12:SET+gRNAs) and CT2H (empty vector as control), in triplicate. Twelve paired-end libraries were sequenced (Illumina^®^ NovaSeq^TM^ system), and a total of 465,471,421 reads (yield 139,641 Mbases; mean quality score = 35.89; and % bases > = 30 = 93.45) were obtained and pre-processed for quality (S5 Table). After extraction of gene hit counts, a gene hit-counts table was used for downstream differential expression analysis between the various samples. High quality sequences were mapped to the *S*. *lycopersicum* genome (*Solanum_lycopersicum*.SL3.0.55.refseq). Differentially expressed genes (DEGs) among the different samples were collected and identified with the help of the DESeq2 package (https://bioconductor.org/). Customary principal component analysis was performed to reveal the similarity within and between groups, hierarchical biclustering analysis showed the homogeneity within the groups and were consistent with PCA and mean-difference plots were calculated to show the log-fold change and average abundance of each gene (Fig 5).

**Fig 6:**
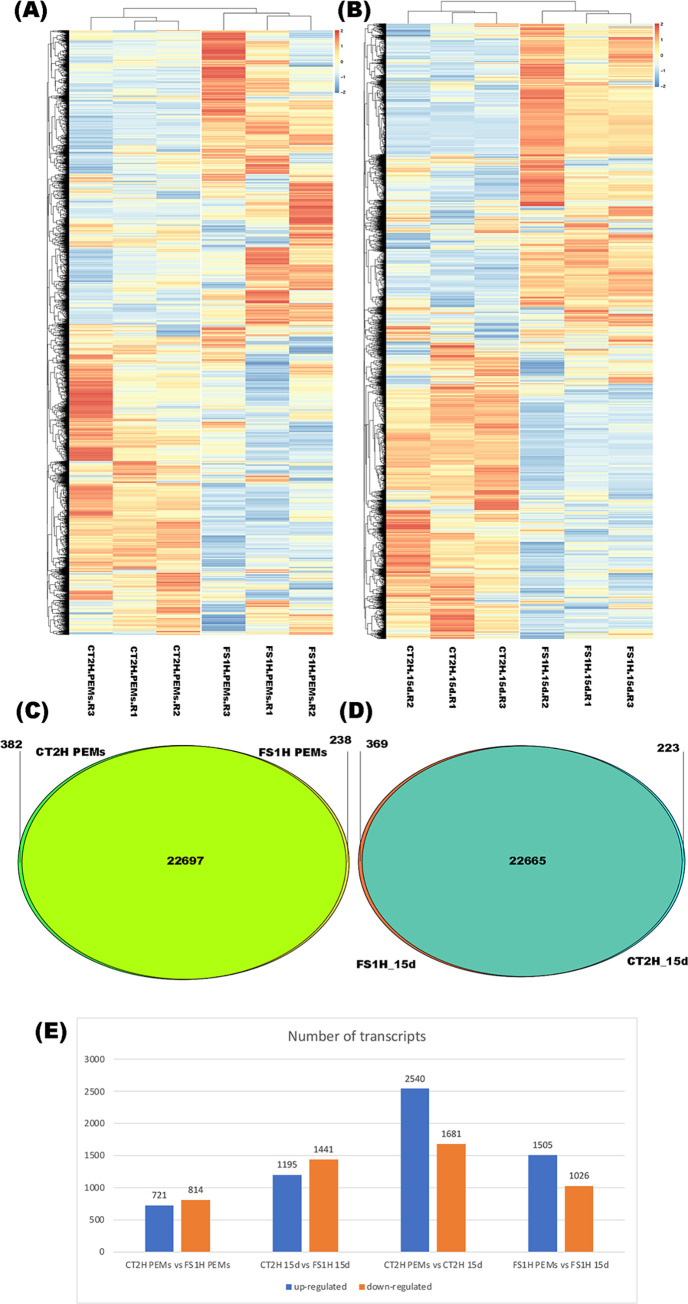
Differential gene expression profile of *Solanum lycopersicum* cv. Micro-Tom. (A). FS1H PEMs. (B) FS1H 15-days somatic embryos (in G9-2iP). A-B: Heatmaps and hierarchical clustering analysis of differentially expressed genes (DEGs). DEGs in each sample were identified with log2(fold-change) of ≥±1. Colors from yellow to red indicate up-regulation; colors from white to blue indicate down-regulation. (C). FS1H PEMs. (D) FS1H 15-days somatic embryos. C-D: Venn diagrams showing shared DEGs among the different samples. Overlapping regions correspond to the number of genes appearing in more than one condition. (E) Number of up-regulated or down-regulated transcripts in each sample.

Next, multiple pairwise comparisons from DEGs (S5 Table) were analyzed. To identify co-regulated genes across the treatment conditions, cluster heatmaps of differentially expressed genes, sorted by their fold-change (log_2_(fold-change) ≥ ±1), were performed (Fig 6A and 6B). As expected, the differential abundance analysis for each transcriptome showed that significant changes occur in the gene expression profiles when the embryos are transferred to G9-2iP medium. Interestingly, most up-regulated genes in FS1H samples were down-regulated in CT2H and up-regulated genes in CT2H were down-regulated FS1H samples. Furthermore, the dynamic transcriptional reprogramming shows a more homogeneous clustering 15 days after the embryos were transferred to G9-2iP medium. Venn diagrams of common and group-specific DEGs compare the two groups and illustrate the number of shared and unique transcripts in PEMs and embryos (15d) (Fig 6C and 6D).

Accordingly, DEGs in each treatment versus the control sample were identified by using the log2(fold-change) of ≥ ± 1 criterium. Based on this condition, from a total of 1535 DEGs between CT2H PEMs and FS1H PEMs, 721 transcripts were up-regulated and 814 were down-regulated. Amongst CT2H embryos (15d) and FS1H embryos (15d), 1195 transcripts were up-regulated and 1441 were down-regulated (total = 2636 DEGs) (Fig 6E).

We selected four genes related to SEs, for follow-up with qPCR. The *FIE* (*Solyc07g064090*.*2*.*1*), *WUSCHEL* (*WUS*, *Solyc02g083950*.*2*.*1*), *FUS3* (*Solyc02g094460*.*1*.*1*) and *LEC1-like* (*Solyc05g005370*.*1*.*1*) genes, with specific expression during PEMs and embryos (15d), were selected as marker genes to evaluate their relative expression by qPCR (S3 Fig). Important to mention is that BlastX analysis of the *Solyc05g005370*.*1*.*1* mRNA sequence against the *Arabidopsis* (TAIR) protein database (at the Sol Genomics Network, https://solgenomics.net/), indicates that the *Solyc05g005370*.*1*.*1* gene is homologous to *AT1G21970* (gene_synonym “AtLEC1; EMB 212; EMB212; EMBRYO DEFECTIVE 212; LEAFY COTYLEDON 1; NF-YB9; NUCLEAR FACTOR Y; SUBUNIT B9; T26F17.20; T26F17_20”). Thus, we labeled it here *SlLEC1*-like. Hence, qPCR analysis showed differential expression levels between PEMs and embryos (15d). *FIE*, *WUS*, *LEC1-like* and *FUS3*, selected as molecular marker genes, were expressed (up-regulated) in PEMs, while they were almost not expressed (down-regulated) during SE development (15d embryos) (S3 Fig). Consequently, qPCR verification demonstrated a high correlation between RNA-seq and qPCR data solely for those four marker genes (in PEMs and SE).

### Hierarchical clustering analysis of pathways or groups of genes involved in cell differentiation and defense mechanisms

To explore key elements in somatic embryogenesis signaling pathways, heatmaps and hierarchical clustering analysis from DEGs were generated according to their functional annotation, among PEMs and 15d embryos. For instance, heatmaps were generated for phytohormone-related genes (auxins, gibberellin, cytokinins, jasmonic acid, salicylic acid, ethylene, abscisic acid), transcriptional regulation (WRKY, nuclear TF-related, MYC, MYB), SET-domain groups (SDG), homeobox-related, MADS box-related, sugar transporter-related, trehalose-related, cytochrome-related, epidermal patterning-related, histone-related, pathogenesis-related and disease resistance-related genes (S4–S6 Figs).

As shown in S4 Fig, heatmaps provide an overview of the different gene expression profiles, of the diverse pathways that were induced. As mentioned, most up-regulated genes in FS1H samples were down-regulated in CT2H and up-regulated genes in CT2H were down-regulated FS1H samples (e.g., auxin response factor, chromatin remodeling proteins; S4A, S4B, S4K and S4L Fig). Also, transcriptional reprogramming shows a more homogeneous clustering in 15d embryos. Consequently, these results suggest that somatic embryo production and maturation in FS1H is attained via distinctive pathways. For example, genes associated with cytokinin signaling were highly expressed in FS1H PEMs or showed gradual increase in expression during FS1H somatic embryogenesis (15d embryos), whereas most of gibberellin oxidases involved in signaling of gibberellic acid (GA) were to a lesser extent expressed in FS1H PEMs, relative to CT2H control (S4C–S4F Fig). In addition, auxin response factors (ARFs) were downregulated in FS1H PEMs (S4 Fig), while AUX/IAA proteins were upregulated (which inhibit transcription of genes activated by ARFs). Furthermore, most genes coding for core histones, histone variants and chromatin remodeling proteins were up-regulated in FS1H PEMs, but down-regulated in FS1H 15 embryos (relative to CT2H control; S4K and S4L Fig).

### Protein-protein interaction network analysis using STRING

Next, to investigate the relationship amongst all identified differentially expressed genes from the RNA-seq analysis and to explore key proteins associated with embryo development and maturation, protein-protein interaction (PPI) and gene ontology analyses were performed using the STRING database (https://string-db.org; based on the *S*. *lycopersicum* genome). Networks of PPIs characterizing gene sets at log2(fold-change) ≥ ± 1 were constructed, and proteins were grouped into functional classes according to their biological processes to establish the potential relationship among DEGs, to identify important network markers, and to gain insight into the molecular mechanisms involved in somatic embryo development.

**Fig 7:**
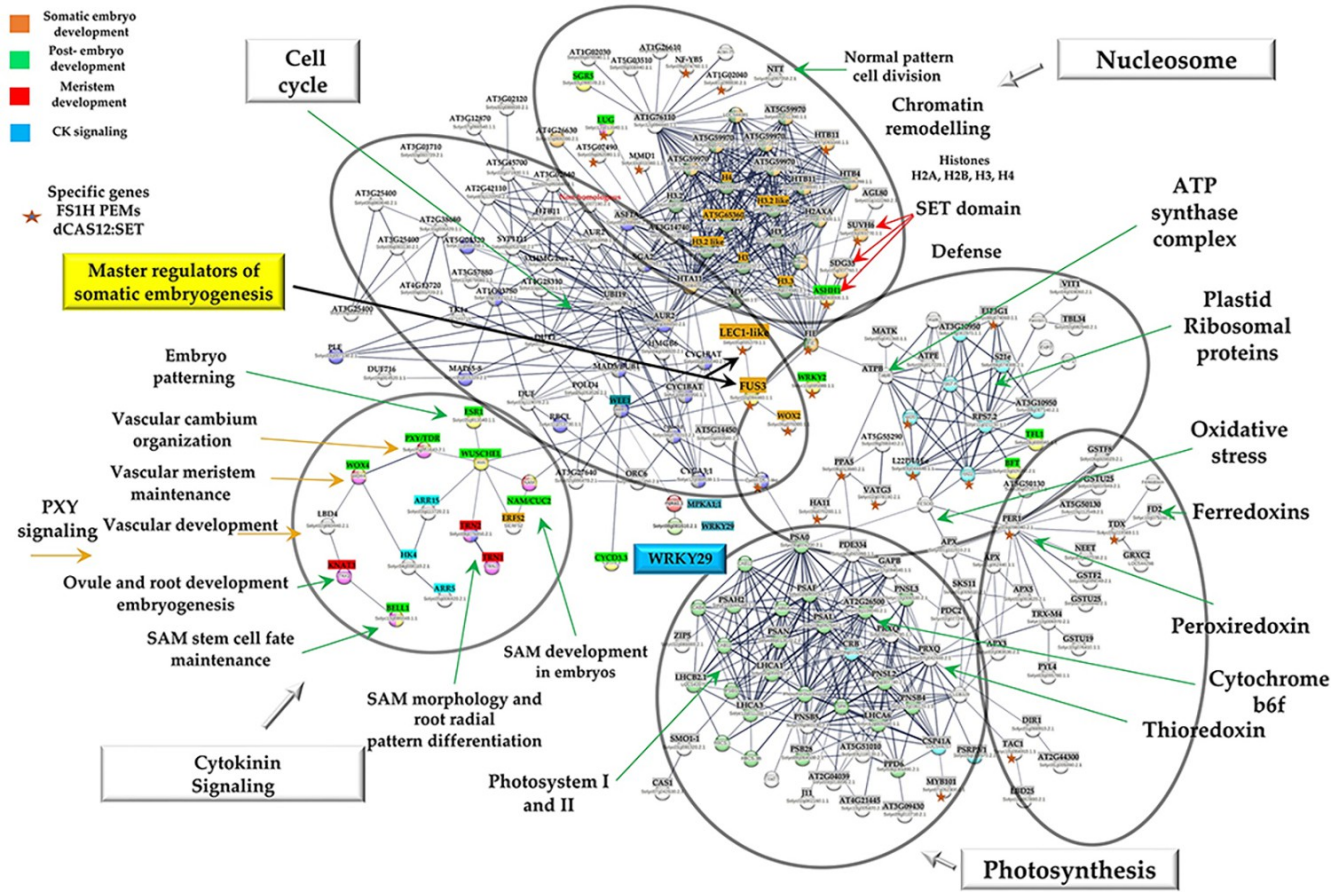
The protein-protein interaction (PPI) network of PEM proteins in tomato revealed by functional protein association networks (STRING) analysis. Protein–protein interaction network of DEGs from FS1H PEMs (interaction score with a 0.6 confidence, without including disconnected nodes), produced by STRING analysis A total of 198 unique homologous proteins from *Solanum lycopersicum* are shown in the PPI network. Six modules are indicated in circles. Module 1: Proteins mainly involved in cytokinin (CK) signaling, stem cell maintenance, embryo patterning, SE development (SAM and RAM embryo formation), and vascular regulation. Module 2: Proteins mainly involved in cell cycle. Module 3: Proteins associated to chromatin remodeling. Module 4: Proteins involved in photosynthesis. Module 5: Ribosomal proteins, proteins important for stress defense and cell wall biogenesis. Module 6: Proteins involved in transcriptional oxidative stress response (e.g. redoxins, peroxidases). Please refer to S4 Table for abbreviations. Specific genes in “FS1H PEMs dCas12:SET” correspond to exclusive raw reads counts, or genes exclusively expressed in this sample (reads only detected in FS1H PEMs; see S6 Table for a list of transcripts).

Predicted PPI networks for PEMs and 15d embryos were generated by using, as input, genes upregulated in FS1H (when compared to CT2H), as well as those exclusively expressed in this sample (exclusive raw reads counts, or transcripts only detected in FS1H; see S6 Table for a list of transcripts). The PPI network for FS1H PEMs is composed of 198 genes, including 26 genes that were specifically expressed in this treatment, where six modules formed tightly connected clusters (using an interaction score with a 0.6 confidence; Fig 7). Module 1 contained proteins mainly involved in cytokinin (CK) signaling, stem cell maintenance, embryo patterning, SE development (SAM and RAM embryo formation), and vascular regulation. Module 2 included those proteins mainly involved in cell cycle. Module 3 contained proteins associated with chromatin remodeling. Module 4 contained proteins involved in photosynthesis. Module 5 included ribosomal proteins, proteins important for stress defense and cell wall biogenesis. Module 6 contained proteins involved in transcriptional oxidative stress response (e.g. redoxins, peroxidases). To shed light on the potential molecular mechanisms involved during PEMs and embryo development, the simple analysis of the PPI network indicates that this stage is mainly regulated by genes involved in CK signaling and embryo patterning (Module 1), as well as in chromatin remodeling (Module 3), and that WRKY29 could interact, directly or indirectly, with those genes or proteins indispensable for embryo development (Fig 7).

**Fig 8:**
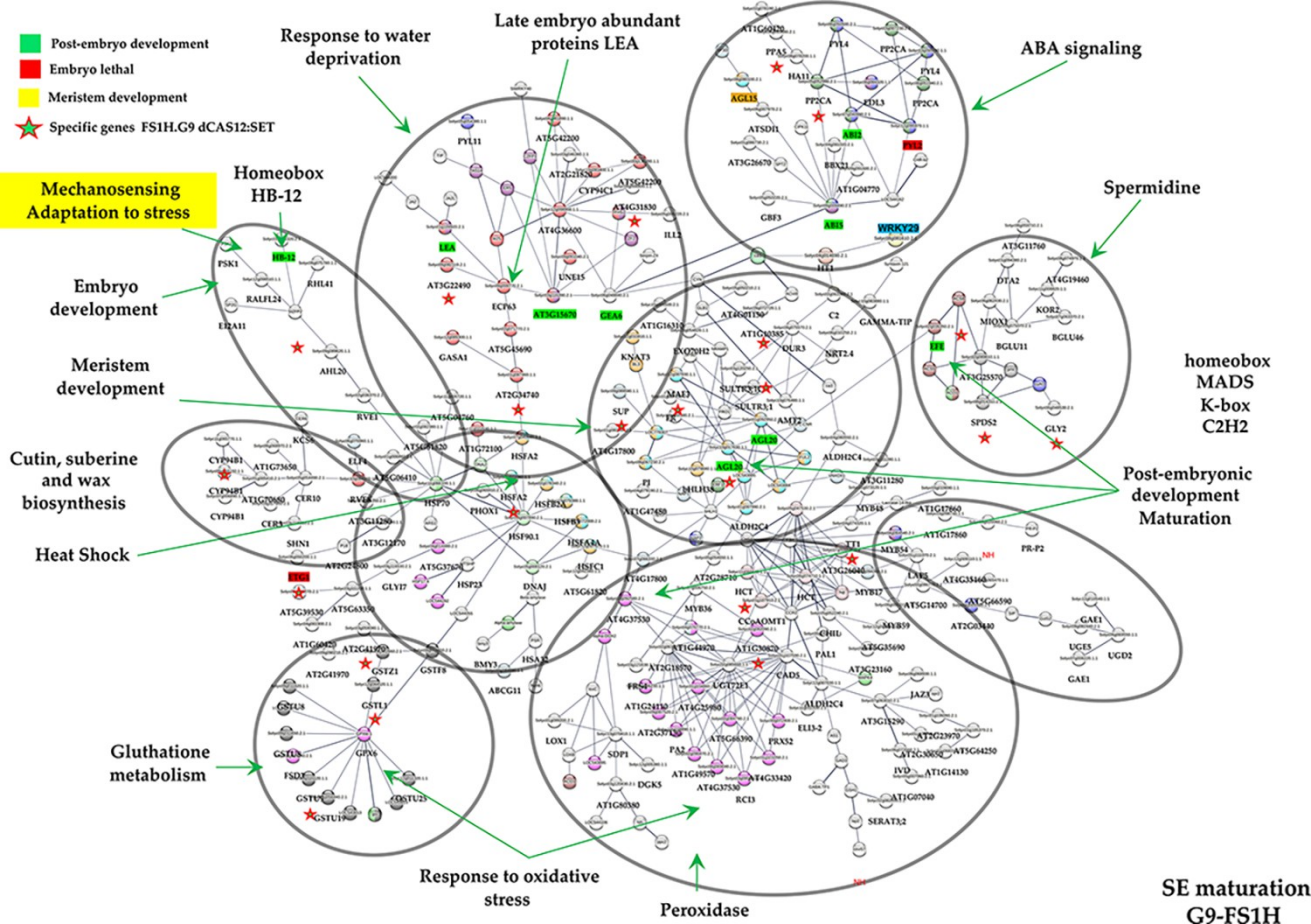
The tomato protein-protein interaction (PPI) network of SE (15d embryos in G9-2iP medium) revealed by functional protein association networks (STRING) analysis. Protein–protein interaction network of DEGs from FS1H 15d embryos (interaction score with a 0.6 confidence, without including disconnected nodes), produced by STRING analysis. A total of 271 unique homologous proteins from *Solanum lycopersicum* are shown. Ten modules are indicated in circles. Module 1: Proteins mainly involved in mechano-sensing and stress-adaptation. Module 2: Heat-shock proteins. Module 3: Proteins related to glutathione metabolism. Module 4: Biopolymers, wax and suberine biosynthesis-related proteins. Module 5: Response to water deprivation and late embryogenesis abundant proteins. Module 6: ABA signaling proteins. Module 7: Proteins involved in stem cell maintenance of meristems (meristem development). Module 8: Phenylpropanoid biosynthesis. Module 9: Carbohydrate metabolism. Module 10: Response to oxidative stress. The PPI network is shown in the confidence view generated by STRING analysis. Please refer to S5 Table for abbreviations. Specific genes in “FS1H.G9 dCas12:SET” correspond to exclusive raw reads counts, or genes exclusively expressed in this sample (reads only detected in FS1H 15-days embryos; see S6 Table for a list of transcripts).

In contrast, FS1H somatic embryos (15d) induced to maturation on medium with high content of gelrite (9g/L, osmotic pressure of − 1.47 MPa; S4 Table), and in the presence of 2-iP as cytokinin, produced a predicted PPI network of 271 proteins, where ten modules formed tightly connected clusters, including 33 genes specifically expressed in this treatment (Fig 8). Module 1 contained proteins mainly involved in mechano-sensing and stress-adaptation. Module 2 included mainly heat-shock proteins. Module 3 contained proteins related to glutathione metabolism. Module 4 is related to biopolymers, wax and suberine biosynthesis. Module 5 includes response to water deprivation and late embryogenesis abundant proteins. Module 6 encompasses ABA signaling proteins. Whereas modules 6 to 10 comprise proteins involved in stem cell maintenance of meristems (meristem development), phenylpropanoid biosynthesis, carbohydrate metabolism, and response to oxidative stress, respectively. Thus, the PPI analysis hints that somatic embryo maturation is largely regulated by genes involved in the process of embryogenesis (e.g., late embryogenesis abundant proteins, LEA proteins; Agamous-like; Arabinogalactan protein 14, AGP14) and post-embryonic development (e.g., ethylene-forming enzyme, EFE, AGL20; AT-hook motif nuclear localized protein 23, AHL23). As shown, the predicted networks suggest that FS1H embryo maturation in BK2iP medium can be achieved by a singular network (in agreement with the heatmaps and hierarchical clustering analysis) (Figs 7 and 8).

## Discussion

In Arabidopsis, osmotic stress induces the formation of somatic embryos as a result of the transfer of explants from an auxin-free medium to an auxin-containing medium [52]. However, when using exogenous auxins, there are different outcomes that make it challenging to detect specific changes related directly to SEs [53]. In carrot, SEs can be induced via culturing explants on phytohormone-free medium containing high amounts of sucrose, followed by transferring the explants to the same type of medium but with lower sucrose concentration [53]. In tomato, somatic embryos have been induced from hypocotyls, intact seedlings, cotyledon explants, etc. However, efficient protocols for a significant propagation of tomato by way of SEs are still missing. Consequently, due to the advance of genome editing technologies, efficient and high-throughput transformation methods are essential for the generation of transgenic and edited lines for basic research studies that can lead to crop improvement [54].

Tomato cv. Micro-Tom is a perfect plant model system for genetic transformation and plant regeneration [44]. A process to increase the efficiency of genetic transformation, genome editing and, ultimately, plant regeneration, is *via* SEs. Somatic embryogenesis (SEs) occurs in nature [16,17], and it has been largely induced in plant tissue culture media using synthetic auxins (e.g., 2,4-dichlorophenoxyacetic acid, also known as 2,4-D; Dicamba; Picloram), and not often by using natural plant hormones like cytokinins. Consequently, we have focused on utilizing an induction method by adopting a combination of osmotic stress treatment and the use of cytokinins.

The use of cytokinins to induce SEs in tomato has been reported. For instance, Newman and colleagues have shown that benzyladenine (also called 6-Benzylaminopurine, or BAP) has a stimulatory effect on the conversion of embryogenic cells into SE [55]. Furthermore, Godishala and colleagues have shown that when auxins are supplemented alone, callus were produced but without any meristematic center; however, an exogenous resource of BAP favors SE development and germination in *Solanum surattense* [51]. Consequently, supplementation of cytokinins can compensate for the detrimental effects of auxins seen on meristem development. Also, the addition of gibberellic acid (also called GA_3_) was required for somatic embryo germination and elongation and promoted maturation and plantlet development [51]. In contrast, Dan and colleagues have used various combinations and concentrations of zeatin riboside and IAA, to regenerate transgenic Micro-Tom plants [56].

Accordingly, a systematic high throughput regeneration technology for tomato has been lacking, particularly for functional genomic applications and (epi)genome editing. Hence, we report a reproducible and simple method for inducing epigenetically edited somatic embryo formation from tomato cotyledonary explants, by a combination of osmotic stress and cytokinin treatment (explant culture on MS-BK2iP osmotic treatment medium 24 h before and 24 h after transformation). Such process enabled us to produce the highest percentage of CRISPRa-edited PEMs per explant (when compared to MS-NAA/BAP conventional osmotic medium, or MS-Zea osmotic treatment medium; Table 1), as well as the highest number of PEMs producing SE. Furthermore, we determined that the predominant morphogenetic pathway for most of the lines was the development of PEMs and SE from cotyledon explants, contrasting with the canonical organogenic pathway characterized by shoot development. The shape of PEMs was rounded and yellowish-green. In addition, the TR2H and FS1H embryogenic lines sub-cultured on MS-BK2iP selective medium produced the highest number of secondary SE per PEM (Fig 1C), capable of plant conversion. For the maturation, rooting and elongation medium, the G9-2iP medium generated shoots producing roots, whereas the G9-Zea medium generated shoots that lacked root formation.

Hence, to categorize genes possibly associated with SE induction and germination, a transcriptome analysis was performed for the CRISPRa-edited FS1H line which produced a great number of embryos in selective media, with the highest *SlWRKY29* expression and enhanced H3K4me3 levels at its 5’-end region (both indicative of efficient CRISPRa), and showing normal development of SAM and RAM in rooting and elongation medium.

As mentioned, *WRKY* genes are involved in the regulation of multiple stress responses, secondary metabolism, as well as in callus development and somatic embryogenesis. For example, in *Arabidopsis* and papaya there is an enhanced expression of *WRKY* genes during SEs [28,57]. Similarly, soybean transcriptome analysis has shown enhanced expression of genes encoding WRKY family transcription factors in cells undergoing de-differentiation during SE induction [29]. Furthermore, expression of the *Panax ginseng PgWRKY6* is upregulated during embryogenic callus development (in response to 2,4-D induction), and functions in the process of SEs in *Panax* species [26]. Correspondingly, our transcriptome analysis shows that not only *WRKY29*, but multiple genes coding for WRKY transcription factors are upregulated in FS1H PEMs (e.g., *WRKY 81*, *WRKY 70*, *WRKY 31*, *WRKY 21*, *WRKY 12*, *WRKY 1*, etc.; S5 Fig), some of which are subsequently downregulated in mature 15d embryos (S5 Fig). Interestingly, the *SlWRKY6* gene homolog remains downregulated in our cytokinin-induced *SlWRKY29* CRISPRa-edited PEMs and 15d embryos (contrary to its 2,4-D induction in ginseng) [26].

Indeed, cytokinin-induced CRISPRa has an effect on auxin signaling since PEMs exhibited downregulation of numerous genes coding for auxin response factors (ARFs), and enhancement of AUX/IAA proteins encoded by the upregulated auxin early response gene family (which inhibit transcription of genes activated by ARFs) [58] (S4A and S4B Fig). Also, genes associated with cytokinin signaling were highly expressed in FS1H PEMs. For instance, there is an increase in the expression of *LONELY GUY* (*LOG*) gene homologs, which code for cytokinin riboside 5′-monophosphate phosphoribohydrolases, involved in the synthesis of isopentenyladenine (iP) and in cytokinin activation [59]. Moreover, in FS1H PEMs, there is enhanced expression of genes coding for enzymes catalyzing the formation of glycosyl conjugates of zeatin (O-glucosyltransferase and O-xylosyltransferase; S4I and S4J Fig), which have a central function in regulating the level of active cytokinins [60,61]. This could contribute to the spatio-temporal distribution of bioactive cytokinins, most likely involved here in cell differentiation, and favors the proposal that cytokinins and the cytokinin signaling pathway, were all primarily involved in cell differentiation and SE formation.

Furthermore, CRISPRa-edited FS1H PEMs exhibited overexpression of morphogenic genes [62], such as *WUS*, *FIE*, *LEC1* and *FUS3* (S3 Fig and S6 and S7 Tables). Accordingly, it has been shown in Arabidopsis that overexpression of the *WUS* gene, for instance, enhances the induction of somatic embryogenesis and improves plant regeneration [62,63]. Thus, *WRKY29* activation enhances a pre-existing embryogenic response [62], heightening the number of PEMs producing SE (Table 1) and the formation of secondary somatic embryos (Fig 1). Further research is necessary to determine whether or not additional overexpressed genes in CRISPRa-edited FS1H PEMs could improve SEs and plant regeneration by activating, or acting as, morphogenic genes.

Moreover, to help identify and discover new biologically significant interactions putatively involved in SE induction and germination, the upregulated DEGs were used to build a protein-protein interaction (PPI) network by employing the STRING-database (Figs 7 and 8). In Arabidopsis, the study of different genes has allowed researchers to recognize key molecular mechanisms of SEs. For instance, several genes are specifically activated or differentially expressed during SEs (e.g., *SERK*, *LEC*, *BBM*, *WUS*, *WOX4*, *FUS3*, etc.) [64]. However, direct activation of *WRKY* genes associated with SE induction, pattern-triggered immunity, or defense priming, via CRISPRa, has not been reported. As shown in Fig 7, our analysis on the PPI network suggests that induction of WRKY29, in CRISPRa-edited FS1H PEMs, could favor an interaction with the mitogen-activated protein kinase 6 MPKA1;1 (*Solyc12g019460*.*1*.*1*) [65]. In the predicted network, MPKA1;1 interacts with the cell cycle regulator WEE1 (*Solyc09g074830*.*3*), which in turn interacts with 10 other proteins present in the cell cycle module. For instance: (a) CYCA3;2 (*Solyc04g078310*.*2*.*1*), expressed in actively dividing tissues such as SAM and RAM and lateral root primordia, with critical functions in meristematic tissues [66]; (b) CYCD3;3 (*Solyc04g078470*.*2*.*1*), essential for normal embryonic development [67]; and (c) CYC1BAT (*Solyc01g009040*.*2*.*1* and *Solyc10g080950*.*1*.*1*), involved in the control of cell cycle progression in eukaryotes [68]. Moreover, the cell cycle regulator WEE1 (*Solyc09g074830*.*3*) is also predicted to interact with members of the CK signaling module via NAM/CUC2 (involved in SAM development in embryogenesis and organ separation) [69], and WUSCHEL (WUS; *Solyc02g083950*.*2*.*1*).

The CK signaling module, composed of 14 genes (S7 Table), includes the cytokinin receptor HK4 (WOL, *Solyc04g008110*.*3*), a sensor for vascular morphogenesis [70,71]; ARR5 (*Solyc05g006420*.*3*), a primary CK responsive gene [71,72]; and *ARR15* (*Solyc03g113720*.*3*), shown to interact with the chlorophyll A/B binding protein 3, ubiquitin-associated (UBA)/TS-N domain-containing protein, zinc finger protein AZF3, and polyketide cyclase/dehydrase/lipid transporter [71]. ARR15 is also predicted to interact with WUS, a protein required to specify stem cell identity in embryo meristems, that promotes the vegetative to embryonic transition [73], involved in organizing the SAM in the embryo and with a crucial role in SE [73–76]. Furthermore, *WUS* expression has a threshold-dependent activation in a switch-like manner to turn on only when sufficient cytokinin levels have accumulated [77]. This could help to explain why *WUS* and type-A ARRs (*ARR15* and *ARR5*) were upregulated in our FS1H PEMs.

In our PPI network, WUS interacts with several proteins involved in SAM and RAM development during embryogenesis (like ESR1, *Solyc05g013540*.*1*) [78], and with proteins involved in vascular meristem maintenance (WOX4) [79], vascular cambium organization (PXY/TDR, *Solyc05g051640*.*2*.*1*) [80], and in vascular development (LBD4, *Solyc02g069440*.*2*.*1*) [81]. Within the CK network, WUS is additionally predicted to interact with KNAT3/TKn3 (*Solyc05g005090*.*3*) [82], and with TRN1 (*Solyc03g112750*.*2*.*1*) and TRN2 (*Solyc08g076850*.*2*.*1*), which are required in SAM morphology and root radial pattern [83,84]. The CK signaling cluster also links, via the predicted WUS-FUS3 protein interaction, to the cell cycle cluster. In turn, FUSCA3 (FUS3, *Solyc02g094460*.*1*.*1*), an AP2/B3-like transcriptional factor, could interact with LEC1-like (a master regulator of SE) and FIE (EMB embryo-lethal). In addition, FUS3 is depicted to interact with WOX2 (WUSCHEL-related HOMEOBOX 2), involved in determination of the apical domain during early embryogenesis and required for the stem cell program in the embryogenic shoot meristem [85]. Interestingly, in the PPI model, FUS3 interacts with the chromatin remodeling module *via* FIE (Fertilization-Independent Endosperm protein), a gene specifically expressed in FS1H PEMs. FIE, a core component of the PRC2 complexes is involved in seed development, vegetative phase transition, meristem activity, the vernalization response [86], and plays a critical role in regulating the differentiation and proliferation of stem cells in the moss gametophyte [87]. Furthermore, the expression in FS1H PEMs of antagonistically acting enzymes (e.g. ATXR1/SDG35, SUVH6/SDG23, FIE, ASHH1; within the chromatin remodeling module) and the accumulation of H3 and H4 histones and histone variants, indicate their significance during embryogenic reprogramming and SE formation. In fact, the hierarchical clustering analysis (S4–S6 Figs) indicates that most proteins belonging to the chromatin remodeling module were specifically upregulated during FSH1 PEM development and SE formation, and then downregulated during somatic embryo maturation.

Conversion of SE to plantlets is a common problem under *in vitro* conditions due to an incomplete development of SAM and RAM, or to defects in the meristem organization during SEs. Abnormalities during embryo development, appearing in somatic embryogenesis experiments, are greatly influenced by components in the growth medium and environmental conditions. Thus, medium composition and *in vitro* environmental conditions such as osmotic components, plant growth regulators (PGR), amino acid sources, culture medium pH, and light intensity can induce changes in the SE phenotype. In most cases, abnormalities in SE are related to the use of the synthetic auxin 2,4-D, which disrupts the endogenous auxin balance and the polar auxin transport, interfering with the embryo apical-basal polarity [88]. Therefore, proper understanding of the shoot and root apical meristem development and physiology will greatly enhance our ability to produce SE of improved quality. Accordingly, in our experiments, somatic embryos derived from edited PEMs were induced to maturation on medium with elevated content of gelrite, in the presence of 2-iP. Under such environmental conditions, water content decreases within the SE [89]. Hence, to determine potential genes involved in embryo development and maturation, we additionally used 15-days old embryos (growing on G9-2iP medium) for RNA-seq analysis. Accordingly, the PPI network analyses suggested that several molecular mechanisms were activated during embryo maturation, which are distributed in 10 modules within the predicted PPI model (Fig 8).

Our results indicate that SE maturation and subsequent conversion to plantlets accompanied the activation of genes involved in stress, mechano-sensing and light tolerance adaptation (mechano-sensing and stress-adaptation module; S8 Table). For instance, the homeobox transcription factor HB-12 (*Solyc01g096320*.*2*.*1*), a master regulator and an essential gene for embryo maturation [90,91], is predicted to interact in the PPI network with the zinc finger protein 2, SlZFP2 (*Solyc07g006880*.*1*.*1*), which involved in the early stages of the mechanoreception pathway and specifically expressed in mechanically stimulated tissues in plants [92]. Other interactors of SlZFP2 include RVE1 (*Solyc02g036370*.*2*.*1*) [93], which in turn interacts with members of the heat-shock proteins module (composed of 30 genes; S7 Table) *via* the HSP70 (*Solyc11g066100*.*1*.*1*) [94]. HSP70 is shown to interact with HSP90.1 (*Solyc03g007890*.*2*.*1*), and according to the PPI model, HSP90.1 interacts, on one side, with HSFC1 (heat stress transcription factor C-1, *Solyc12g007070*.*1*.*1*) and a NAC domain protein (*Solyc07g066330*.*2*.*1*) networking with *Solyc02g092580*.*2*.*1* (a peroxidase protein), which links the heat shock module to the response to oxidative stress/peroxidase module); and, on the other, with HSFA2 (*Solyc02g072060*.*1*.*1* and *Solyc02g040680*.*1*.*1*), which functions as the link with the late embryogenesis abundant proteins/LEA module. The NAC domain protein (*Solyc07g066330*.*2*.*1*) is predicted to connect with bHLH1 protein, which functions as a hub in the stem cell maintenance and meristem development module (S7 Table), Interestingly, SPL3 (or LeSPL-CNR), which encodes a member of the SPL (squamosa-promoter binding protein-like) gene family, involved in regulation of flowering and vegetative phase change, networks at the spermidine module with the EFE protein (*Solyc02g036350*.*2*.*1*; 1-aminocyclopropane-1-carboxylate oxidase, or ethylene-forming enzyme), the enzyme that produces the volatile plant hormone ethylene, which regulates many plant developmental processes and stress responses [95,96]. In turn, EFE is foreseen to interact with the 1-aminocyclopropane-1-carboxylate synthases 5 and 8 (ACS5, *Solyc04g077410*.*2*.*1*; and ACS8, *Solyc03g043890*.*2*.1), which catalyze the synthesis of 1-aminocyclopropane-1-carboxylic acid (ACC), a precursor for ethylene. Thus, we hypothesized that in tomato, production of the natural plant hormone ethylene, during embryo maturation, helps regulate plant development, growth, and stress responses.

Altogether, various transcription factors have been recognized to induce SEs when ectopically expressed [97,98]. This indicates that many independent and/or interrelated pathways, as well as numerous regulatory factors, are involved in the acquisition of embryogenic competence during somatic embryogenesis. Furthermore, loss-of-function of specific chromatin remodelers can also induce SEs, revealing that epigenetic mechanisms are likely to be involved in cell dedifferentiation, cell fate, and embryogenic cell formation [99]. Accordingly, ectopic expression of *SlWRKY29* via CRISPR-activation has a beneficial morphogenic growth response by enhancing the formation of SE under in vitro culture conditions (most likely by activation of morphogenic genes; S3 Fig and S6 and S7 Tables). Thus, CRISPRa-edited *SlWRKY29* embryogenic lines could be transformed with candidate genes involved in embryogenic competence, or explants might be transformed with sgRNAs targeting multiple promoters and co-activated together with *SlWRKY29* for accelerated regeneration of edited plants [100].

Nonetheless, efficient employment of species-specific morphogenic genes encounters diverse methodological challenges (e.g. arduous processes of cloning, functional validation, co-expression of combinations of genes) [100]. In addition, when using the CaMV 35S promoter, undesired pleiotropic effects and harmful growth defects could be caused by constitutive expression of morphogenic regulators, which in turn requires to control expression of the morphogenic gene (via inducible expression, developmentally regulated expression, or by excision of the exogenous DNA) to regenerate normal-phenotype plants [62]. An advantage of utilizing CRISPRa is that ectopic gene expression is achieved by making use of the cell’s native apparatus to upregulate target endogenous gene expression levels. Moreover, in CRISPRa a second copy of the target gene is not being introduced into the plant genome. In our experiments, CRISPRa enabled *SlWRKY29* expression without induction of growth defects in the regenerated plants. Further research is required to determine if the edited plants display, for instance, Mendelian inheritance of the epigenetic modification, enhanced disease resistance [30,32,33], augmented ethylene production [31], or tolerance to abiotic stress [101].

### Conclusion

Current research let us hypothesize that CRISPRa-mediated transcriptional activation of *SlWRKY29*, a gene associated with plant immunity and likely involved in somatic embryogenesis, enhance SE formation and germination in the presence of cytokinins and osmotic stress. Accordingly, we have demonstrated that our results are consistent with the premise that directed H3K4me3 methylation marks to the promoter region of *SlWRKY29*, via CRISPRa, establishes a transcriptionally permissive chromatin state and enhances its transcriptional activation, and improves in vitro the formation of SE and regeneration efficiency. Furthermore, we have shown that, under our experimental conditions, the CRISPRa system can be effectively used for greater gene activation when a single effector domain, like the SET-domain, is fused at the C-end of the dCas12 nuclease (dCas12:SET, or FS1H), as well as for higher number of somatic embryo production and plantlet regeneration, when compared to the control constructs. Consequently, enhanced embryo induction and maturation are influenced by the transcriptional effector used, as well as by the medium composition (BK2iP medium) and *in vitro* environmental conditions such as osmotic components, plant growth regulators (auxin-free, cytokinin-rich media), and light intensity. Taken together, our findings suggest that cytokinin supplementation and the activation of *SlWRKY29*, induce variations in the homeostasis of plant hormones, mainly CKs and auxins, which in turn could lead to the expression of multiple transcription factors and enable cell cycle activation, cell differentiation, and SE formation in FS1H PEMs. Furthermore, cytokinin supplementation compensates for the detrimental effects often seen with the use of the synthetic auxin 2,4-D. At the same time, activation of different H3 isoforms and histone-methyl transferases may allow turnover and epigenetic modulation to activate the formation of somatic embryos. In turn, activation of *SlWRKY29* in FS1H 15d embryos is hypothesized to favor the germination of individual Micro-Tom embryos possibly through its link with HT1, which in turn could signal key genes in the process of meristem development. Hence, the extensive range of regulatory factors involved in acquiring embryogenic competence indicates that numerous independent and interrelated pathways are functional in this process. Whether and how these genes work together to promote embryogenic cell formation is still debatable. It is possible that epigenetic changes can regulate cell fate, creating a layer of control superimposed on the activity of the numerous transcription factors that are involved in this process.

## Supporting information

S1 Fig Expression cassettes comprised in the Gateway-assembled plasmids. (A) dCas9S2+gRNA or TR2H vector, containing two SET domains fused each to dCas9 and MS2 protein. It also includes the sgRNA cassette containing three WRKY29 sgRNA’s each under the control of AtU6 promoter and AtU6 terminator. (B) RZ0H same as previous plasmids, but without expressing the sgRNA cassette (used as control). (C) FS1H, with a single SET domain fused to dCas12, and expressing an array of three WRKY29 crRNA’s units under the control of AtU6 promoter and AtU6 terminator. (D) SL0H, same as previous plasmid without expressing the respective crRNA cassette, as control. (E) CT2H, empty vector employed as control. All described plasmids contain a hygromycin resistance cassette as selection marker. For a detailed description of vector construction see S1 Table.
(TIF)

S2 Fig Illustrative description of somatic embryogenesis. Individual transformation events sub-cultured onto fresh selective medium to evaluate the development of normal and anomalous structures. (A) Eight-days old cotyledons after biolistic treatment. (B) Induction of pro-embryogenic masses and development of somatic embryo-like structures growing on MS-BK2iP selective medium. (C) Primary selection on medium containing 9.6 mg/L of Hygromycin. (D) Isolation and selection of embryo-like events. (E) Close-up of (D). (F) Induction of secondary embryogenesis, for dissected and individually sub-cultured embryos onto fresh selective medium, to obtain embryogenic lines. (G) Genotyping of embryogenic lines. The presence of the transgene was confirmed by PCR with 35SCaMV, dCas9 and dCas12 specific primers. All embryogenic lines were genotyped (a few of the positive embryogenic lines are shown). Amplification of the *SlLSM7* gene (endogenous gene) was used as control for the PCR reactions (for a list of primers used see S2 Table).
(TIF)

S3 Fig Transcript levels of the *S*. *lycopersicum FIE*, *WUS*, *LEC1*, *FUS3* gene homologs in edited FS1H PEMs and 15-days embryos. Samples were taken from pro-embryogenic masses (PEMs) and 15-days somatic embryos (in G9-2iP), and the relative expression was determined by qPCR. Data were normalized to the *SlLSM7* reference gene (based on the 2^−ΔΔCT^ method) [48]. Data represent mean ± SD from three independent experiments (n  =  3). Statistical significance was determined with an unpaired two-tailed Student’s t-test (*p < 0.05, *** p < 0.001).
(TIF)

S4 Fig Differential gene expression profiles in FS1H PEM and 15-days somatic embryos (in G9-2iP). Heatmaps providing an overview on gene expression profiles, of the diverse pathways that were induced, based on the log2(fold-change) of ≥±1. Colors from yellow to red indicate up-regulation; colors from white to blue indicate down-regulation.
(TIF)

S5 Fig Differential gene expression profiles in FS1H PEMs and 15-days somatic embryos (in G9-2iP). Heatmaps, providing an overview on gene expression profiles, of the diverse pathways that were induced, based on the log2(fold-change) of ≥±1. Colors from yellow to red indicate up-regulation; colors from white to blue indicate down-regulation.
(TIF)

S6 Fig Differential gene expression profiles in FS1H PEMs and 15-days somatic embryos (in G9-2iP). Heatmaps, providing an overview on gene expression profiles, of the diverse pathways that were induced, based on the log2(fold-change) of ≥±1. Colors from yellow to red indicate up-regulation; colors from white to blue indicate down-regulation.
(TIF)

S1 Table Methods. Vector construction.
(DOCX)

S2 Table DNA sequences. (A) Selected target sequences at the *SlWRKY29* promoter region for dCas9 and dCas12. Highlighted sequences correspond to the PAM sequence. (B) Oligonucleotides used for vector construction. (C) *SlWRKY29* gRNA oligonucleotide cloning reference. (D) Linker sequence in the p143-L2 plasmid and synthetic DNA with the crRNAs (crRNA array) for dCas12. (E) List of oligos used for qPCR. (F) List of oligos used for PCR-genotyping.
(DOCX)

S3 Table Recombination reactions. Recombination reactions between destination and entry vectors to generate the CRISPR-Act2.0 and CRISPR-dCas12 expression vectors used to transform tomato explants via biolistics.
(DOCX)

S4 Table Media. (A) Osmotic treatment medium. (B) Conventional medium. (C) Selective medium. (D) Maturation, rooting and elongation medium.
(DOCX)

S5 Table RNA-seq libraries. (A) Sample sequencing statistics of RNA-seq libraries of S. lycopersicum transcriptome. (B) Pairwise comparisons.
(DOCX)

S6 Table List of transcripts that were exclusively detected in the FS1H samples (PEMs and 15-days embryos in G9-2iP). (DOCX)

S7 Table List of proteins from *Solanum lycopersicum* (FS1H PEM) used to build the PPI network. (XLSX)

S8 Table List of proteins from Solanum lycopersicum (FS1H 15-days embryos) used to build the PPI network. (XLSX)

S1 Raw images (TIF)

## References

[1] Schmidt SM, Belisle M, Frommer WB (2020) The evolving landscape around genome editing in agriculture: Many countries have exempted or move to exempt forms of genome editing from GMO regulation of crop plants. EMBO Rep. doi: 10.15252/embr.202050680 32431018 PMC7271327

[2] Ishii M, Ishii T (2022) Proving that a genome-edited organism is not GMO. Trends Biotechnol 40:525–528. doi: 10.1016/j.tibtech.2021.11.001 34893376

[3] Feng Z, Zhang B, Ding W, et al (2013) Efficient genome editing in plants using a CRISPR/Cas system. Cell Res 23:1229–1232. doi: 10.1038/cr.2013.114 23958582 PMC3790235

[4] Ishino Y, Krupovic M, Forterre P (2018) History of CRISPR-Cas from Encounter with a Mysterious Repeated Sequence to Genome Editing Technology. J Bacteriol. doi: 10.1128/JB.00580-17 29358495 PMC5847661

[5] Jinek M, Chylinski K, Fonfara I, Hauer M, Doudna JA, Charpentier E (2012) A Programmable Dual-RNA–Guided DNA Endonuclease in Adaptive Bacterial Immunity. Science (1979) 337:816–821. doi: 10.1126/science.1225829 22745249 PMC6286148

[6] Agarwal G, Kudapa H, Ramalingam A, et al (2020) Epigenetics and epigenomics: underlying mechanisms, relevance, and implications in crop improvement. Funct Integr Genomics 20:739–761. doi: 10.1007/s10142-020-00756-7 33089419

[7] Kungulovski G, Jeltsch A (2016) Epigenome Editing: State of the Art, Concepts, and Perspectives. Trends Genet 32:101–113. doi: 10.1016/j.tig.2015.12.001 26732754

[8] Thakore PI, Black JB, Hilton IB, Gersbach CA (2016) Editing the epigenome: technologies for programmable transcription and epigenetic modulation. Nat Methods 13:127–137. doi: 10.1038/nmeth.3733 26820547 PMC4922638

[9] Jinek M, Chylinski K, Fonfara I, Hauer M, Doudna JA, Charpentier E (2012) A programmable dual-RNA-guided DNA endonuclease in adaptive bacterial immunity. Science 337:816–821. doi: 10.1126/science.1225829 22745249 PMC6286148

[10] Perez-Pinera P, Kocak DD, Vockley CM, et al (2013) RNA-guided gene activation by CRISPR-Cas9-based transcription factors. Nat Methods 10:973–976. doi: 10.1038/nmeth.2600 23892895 PMC3911785

[11] Zuo Z, Liu J (2017) Structure and Dynamics of Cas9 HNH Domain Catalytic State. Sci Rep. doi: 10.1038/s41598-017-17578-6 29222528 PMC5722908

[12] Gilbert LA, Larson MH, Morsut L, et al (2013) CRISPR-mediated modular RNA-guided regulation of transcription in eukaryotes. Cell 154:442. doi: 10.1016/j.cell.2013.06.044 23849981 PMC3770145

[13] Roca Paixão JF, Gillet FX, Ribeiro TP, Bournaud C, Lourenço-Tessutti IT, Noriega DD, et al (2019) Improved drought stress tolerance in Arabidopsis by CRISPR/dCas9 fusion with a Histone AcetylTransferase. Sci Rep. doi: 10.1038/s41598-019-44571-y 31147630 PMC6542788

[14] García-Murillo L, Valencia-Lozano E, Priego-Ranero NA, Cabrera-Ponce JL, Duarte-Aké FP, Vizuet-de-Rueda JC, et al (2023) CRISPRa-mediated transcriptional activation of the SlPR-1 gene in edited tomato plants. Plant Sci. doi: 10.1016/j.plantsci.2023.111617 36731748

[15] Liu C, Moschou PN (2018) Phenotypic novelty by CRISPR in plants. Dev Biol 435:170–175. doi: 10.1016/j.ydbio.2018.01.015 29402392

[16] Dodeman VL, Ducreux G, Kreis M (1997) REVIEW ARTICLE: Zygotic embryogenesis versus somatic embryogenesis. J Exp Bot 48:1493–1509.

[17] Garcês HMP, Champagne CEM, Townsley BT, Park S, Malhó R, Pedroso MC, et al (2007) Evolution of asexual reproduction in leaves of the genus Kalanchoë. Proc Natl Acad Sci U S A 104:15578–15583. 17893341 10.1073/pnas.0704105104 PMC2000513

[18] Ali D, Alarifi S, Pandian A (2021) Somatic embryogenesis and in vitro plant regeneration of Bacopa monnieri (Linn.) Wettst., a potential medicinal water hyssop plant. Saudi J Biol Sci 28:353–359. doi: 10.1016/j.sjbs.2020.10.013 33424317 PMC7783628

[19] Brooks C, Nekrasov V, Lipppman ZB, Van Eck J (2014) Efficient gene editing in tomato in the first generation using the clustered regularly interspaced short palindromic repeats/CRISPR-associated9 system. Plant Physiol 166:1292–1297. doi: 10.1104/pp.114.247577 25225186 PMC4226363

[20] Duclercq J, Sangwan-Norreel B, Catterou M, Sangwan RS (2011) De novo shoot organogenesis: from art to science. Trends Plant Sci 16:597–606. doi: 10.1016/j.tplants.2011.08.004 21907610

[21] Firoozabady E, Moy Y (2004) Regeneration of pineapple plants via somatic embryogenesis and organogenesis. In Vitro Cellular & Developmental Biology—Plant 40:67–74.

[22] Long Y, Yang Y, Pan G, Shen Y (2022) New Insights Into Tissue Culture Plant-Regeneration Mechanisms. Front Plant Sci. doi: 10.3389/fpls.2022.926752 35845646 PMC9280033

[23] Etienne H, Bertrand B, Georget F, Lartaud M, Montes F, Dechamp E, et al (2013) Development of coffee somatic and zygotic embryos to plants differs in the morphological, histochemical and hydration aspects. Tree Physiol 33:640–653. doi: 10.1093/treephys/tpt034 23729274

[24] Liu G, Zhang D, Zhao T, Yang H, Jiang J, Li J, et al (2022) Genome-wide analysis of the WRKY gene family unveil evolutionary history and expression characteristics in tomato and its wild relatives. Front Genet. doi: 10.3389/fgene.2022.962975 36186453 PMC9520452

[25] Phukan UJ, Jeena GS, Shukla RK (2016) WRKY Transcription Factors: Molecular Regulation and Stress Responses in Plants. Front Plant Sci. doi: 10.3389/fpls.2016.00760 27375634 PMC4891567

[26] Yang Y, Wang N, Zhao S (2020) Functional characterization of a WRKY family gene involved in somatic embryogenesis in Panax ginseng. Protoplasma 257:449–458. doi: 10.1007/s00709-019-01455-2 31760482

[27] Martínez-Aguilar K, Ramírez-Carrasco G, Hernández-Chávez JL, Barraza A, Alvarez-Venegas R (2016) Use of BABA and INA As Activators of a Primed State in the Common Bean (Phaseolus vulgaris L.). Front Plant Sci. 10.3389/FPLS.2016.00653. PMC4870254 27242854

[28] Jamaluddin ND, Mohd Noor N, Goh HH (2017) Genome-wide transcriptome profiling of Carica papaya L. embryogenic callus. Physiol Mol Biol Plants 23:357–368. doi: 10.1007/s12298-017-0429-8 28461724 PMC5391361

[29] Perry SE, Zheng Q, Zheng Y (2016) Transcriptome analysis indicates that GmAGAMOUS-Like 15 may enhance somatic embryogenesis by promoting a dedifferentiated state. Plant Signal Behav. doi: 10.1080/15592324.2016.1197463 27302197 PMC4991326

[30] Sarowar S, Alam ST, Makandar R, Lee H, Trick HN, Dong Y, et al (2019) Targeting the pattern-triggered immunity pathway to enhance resistance to Fusarium graminearum. Mol Plant Pathol 20:626–640. doi: 10.1111/mpp.12781 30597698 PMC6637896

[31] Wang Z, Wei X, Wang Y, Sun M, Zhao P, Wang Q, et al (2023) WRKY29 transcription factor regulates ethylene biosynthesis and response in arabidopsis. Plant Physiol Biochem 194:134–145. doi: 10.1016/j.plaphy.2022.11.012 36403487

[32] Jaskiewicz M, Conrath U, Peterhälnsel C (2011) Chromatin modification acts as a memory for systemic acquired resistance in the plant stress response. EMBO Rep 12:50–55. doi: 10.1038/embor.2010.186 21132017 PMC3024125

[33] Conrath U, Beckers GJM, Langenbach CJG, Jaskiewicz MR (2015) Priming for enhanced defense. Annu Rev Phytopathol 53:97–119. doi: 10.1146/annurev-phyto-080614-120132 26070330

[34] Lowder LG, Zhang D, Baltes NJ, Paul JW, Tang X, Zheng X, et al (2015) A CRISPR/Cas9 Toolbox for Multiplexed Plant Genome Editing and Transcriptional Regulation. Plant Physiol 169:971–985. doi: 10.1104/pp.15.00636 26297141 PMC4587453

[35] Lowder LG, Paul JW, Qi Y (2017) Multiplexed Transcriptional Activation or Repression in Plants Using CRISPR-dCas9-Based Systems. Methods Mol Biol 1629:167–184. 28623586 10.1007/978-1-4939-7125-1_12

[36] Lowder LG, Zhou J, Zhang Y, Malzahn A, Zhong Z, Hsieh TF, et al (2018) Robust Transcriptional Activation in Plants Using Multiplexed CRISPR-Act2.0 and mTALE-Act Systems. Mol Plant 11:245–256. doi: 10.1016/j.molp.2017.11.010 29197638

[37] Lei Y, Lu L, Liu HY, Li S, Xing F, Chen LL (2014) CRISPR-P: a web tool for synthetic single-guide RNA design of CRISPR-system in plants. Mol Plant 7:1494–1496. doi: 10.1093/mp/ssu044 24719468

[38] Labun K, Montague TG, Krause M, Torres Cleuren YN, Tjeldnes H, Valen E (2019) CHOPCHOP v3: expanding the CRISPR web toolbox beyond genome editing. Nucleic Acids Res 47:W171–W174. doi: 10.1093/nar/gkz365 31106371 PMC6602426

[39] Chumpookam J, Lin HL, Shiesh CC (2012) Effect of Smoke-water on Seed Germination and Seedling Growth of Papaya (Carica papaya cv. Tainung No. 2). HortScience 47:741–744.

[40] Murashige T, Skoog F (1962) A Revised Medium for Rapid Growth and Bio Assays with Tobacco Tissue Cultures. Physiol Plant 15:473–497.

[41] Silva JAT da (2004) The effect of carbon source on in vitro organogenesis of chrysanthemum thin cell layers. Bragantia 63:165–177.

[42] Yaseen M, Ahmad T, Sablok G, Standardi A, Hafiz IA (2013) Review: role of carbon sources for in vitro plant growth and development. Mol Biol Rep 40:2837–2849. doi: 10.1007/s11033-012-2299-z 23212616

[43] Shikata M, Ezura H (2016) Micro-Tom Tomato as an Alternative Plant Model System: Mutant Collection and Efficient Transformation. pp 47–55. 10.1007/978-1-4939-3115-6_5 26577780

[44] Pino LE, Lombardi-Crestana S, Azevedo MS, Scotton DC, Borgo L, Quecini V, et al (2010) The Rg1 allele as a valuable tool for genetic transformation of the tomato “Micro-Tom” model system. Plant Methods. doi: 10.1186/1746-4811-6-23 20929550 PMC2958934

[45] Cabrera-Ponce JL, López L, Assad-Garcia N, Medina-Arevalo C, Bailey AM, Herrera-Estrella L (1997) An efficient particle bombardment system for the genetic transformation of asparagus (Asparagus officinalis L.). Plant Cell Rep 16:255–260. doi: 10.1007/BF01088276 30727658

[46] Müller OA, Grau J, Thieme S, Prochaska H, Adlung N, Sorgatz A, et al (2015) Genome-Wide Identification and Validation of Reference Genes in Infected Tomato Leaves for Quantitative RT-PCR Analyses. PLoS One. doi: 10.1371/journal.pone.0136499 26313760 PMC4552032

[47] Expósito-Rodríguez M, Borges AA, Borges-Pérez A, Pérez JA (2008) Selection of internal control genes for quantitative real-time RT-PCR studies during tomato development process. BMC Plant Biol. doi: 10.1186/1471-2229-8-131 19102748 PMC2629474

[48] Livak KJ, Schmittgen TD (2001) Analysis of relative gene expression data using real-time quantitative PCR and the 2(-Delta Delta C(T)) Method. Methods 25:402–408. doi: 10.1006/meth.2001.1262 11846609

[49] Solomon ER, Caldwell KK, Allan AM (2021) A novel method for the normalization of ChIP-qPCR data. MethodsX. doi: 10.1016/j.mex.2021.101504 34754775 PMC8563474

[50] Love MI, Huber W, Anders S (2014) Moderated estimation of fold change and dispersion for RNA-seq data with DESeq2. Genome Biol. doi: 10.1186/s13059-014-0550-8 25516281 PMC4302049

[51] "Godishala V "Mangamoori LN "Nanna A (2011) Plant regeneration via somatic embryogenesis in cultivated tomato (Solanum lycopersicum L.). Journal of Cell and Tissue Research 11:2521–2528.

[52] Ikeda-Iwai M, Umehara M, Satoh S, Kamada H (2003) Stress-induced somatic embryogenesis in vegetative tissues of Arabidopsis thaliana. Plant J 34:107–114. doi: 10.1046/j.1365-313x.2003.01702.x 12662313

[53] Kamada H, Ishikawa K, Saga H, Harada H (1993) Induction of Somatic Embryogenesis in Carrot by Osmotic Stress. Original Papers Plant Tissue Culture Letters 10:38–44.

[54] Van Eck J, Keen P, Tjahjadi M (2019) Agrobacterium tumefaciens-Mediated Transformation of Tomato. Methods Mol Biol 1864:225–234. doi: 10.1007/978-1-4939-8778-8_16 30415340

[55] Newman PO, Krishnaraj S, Saxena PK (1996) Regeneration of Tomato (Lycopersicon esculentum Mill.): Somatic Embryogenesis and Shoot Organogenesis from Hypocotyl Explaints Induced with 6-Benzyladenine. https://doi.org/101086/297375157:554–560.

[56] Dan Y, Yan H, Munyikwa T, Dong J, Zhang Y, Armstrong CL (2006) MicroTom—a high-throughput model transformation system for functional genomics. Plant Cell Rep 25:432–441. doi: 10.1007/s00299-005-0084-3 16341726

[57] Gliwicka M, Nowak K, Balazadeh S, Mueller-Roeber B, Gaj MD (2013) Extensive modulation of the transcription factor transcriptome during somatic embryogenesis in Arabidopsis thaliana. PLoS One. doi: 10.1371/journal.pone.0069261 23874927 PMC3714258

[58] Luo J, Zhou JJ, Zhang JZ (2018) Aux/IAA Gene Family in Plants: Molecular Structure, Regulation, and Function. Int J Mol Sci. doi: 10.3390/ijms19010259 29337875 PMC5796205

[59] Kuroha T, Tokunaga H, Kojima M, Ueda N, Ishida T, Nagawa S, et al (2009) Functional analyses of LONELY GUY cytokinin-activating enzymes reveal the importance of the direct activation pathway in Arabidopsis. Plant Cell 21:3152–3169. doi: 10.1105/tpc.109.068676 19837870 PMC2782294

[60] Martin RC, Mok MC, Mok DWS (1999) A gene encoding the cytokinin enzyme zeatin O-xylosyltransferase of Phaseolus vulgaris. Plant Physiol 120:553–557. doi: 10.1104/pp.120.2.553 10364407 PMC59294

[61] Mok DWS, Mok MC (2001) CYTOKININ METABOLISM AND ACTION. Annu Rev Plant Physiol Plant Mol Biol 52:89–118. doi: 10.1146/annurev.arplant.52.1.89 11337393

[62] Gordon-Kamm B, Sardesai N, Arling M, Lowe K, Hoerster G, Betts S, et al (2019) Using Morphogenic Genes to Improve Recovery and Regeneration of Transgenic Plants. Plants 8:38. doi: 10.3390/plants8020038 30754699 PMC6409764

[63] Bouchabké-Coussa O, Obellianne M, Linderme D, Montes E, Maia-Grondard A, Vilaine F, et al (2013) Wuschel overexpression promotes somatic embryogenesis and induces organogenesis in cotton (Gossypium hirsutum L.) tissues cultured in vitro. Plant Cell Rep 32:675–686. doi: 10.1007/s00299-013-1402-9 23543366

[64] Kumar V, Van Staden J (2017) New insights into plant somatic embryogenesis: an epigenetic view. Acta Physiologiae Plantarum 2017 39:9 39:1–17.

[65] Asai T, Tena G, Plotnikova J, Willmann MR, Chiu WL, Gomez-Gomez L, et al (2002) MAP kinase signalling cascade in Arabidopsis innate immunity. Nature 415:977–983. doi: 10.1038/415977a 11875555

[66] Takahashi I, Kojima S, Sakaguchi N, Umeda-Hara C, Umeda M (2010) Two Arabidopsis cyclin A3s possess G1 cyclin-like features. Plant Cell Rep 29:307–315. doi: 10.1007/s00299-010-0817-9 20130883

[67] Xu X, Chen X, Chen Y, Zhang Q, Su L, Chen X, et al (2020) Genome-wide identification of miRNAs and their targets during early somatic embryogenesis in Dimocarpus longan Lour. Sci Rep. doi: 10.1038/s41598-020-60946-y 32170163 PMC7069941

[68] Day IS, Reddy AS, Golovkin M (1996) Isolation of a new mitotic-like cyclin from Arabidopsis: complementation of a yeast cyclin mutant with a plant cyclin. Plant Mol Biol 30:565–575. doi: 10.1007/BF00049332 8605306

[69] Takada S, Hibara KI, Ishida T, Tasaka M (2001) The CUP-SHAPED COTYLEDON1 gene of Arabidopsis regulates shoot apical meristem formation. Development 128:1127–1135. doi: 10.1242/dev.128.7.1127 11245578

[70] Mähönen AP, Bonke M, Kauppinen L, Riikonen M, Benfey PN, Helariutta Y (2000) A novel two-component hybrid molecule regulates vascular morphogenesis of the Arabidopsis root. Genes Dev 14:2938–2943. doi: 10.1101/gad.189200 11114883 PMC317089

[71] Dortay H, Gruhn N, Pfeifer A, Schwerdtner M, Schmülling T, Heyl A (2008) Toward an interaction map of the two-component signaling pathway of Arabidopsis thaliana. J Proteome Res 7:3649–3660. doi: 10.1021/pr0703831 18642946

[72] D’Agostino IB, Deruère J, Kieber JJ (2000) Characterization of the response of the Arabidopsis response regulator gene family to cytokinin. Plant Physiol 124:1706–1717. doi: 10.1104/pp.124.4.1706 11115887 PMC59868

[73] Zuo J, Niu QW, Frugis G, Chua NH (2002) The WUSCHEL gene promotes vegetative-to-embryonic transition in Arabidopsis. Plant J 30:349–359. doi: 10.1046/j.1365-313x.2002.01289.x 12000682

[74] Laux T, Mayer KFX, Berger J, Jürgens G (1996) The WUSCHEL gene is required for shoot and floral meristem integrity in Arabidopsis. Development 122:87–96. doi: 10.1242/dev.122.1.87 8565856

[75] Meng Y, Liu H, Wang H, et al (2019) HEADLESS, a WUSCHEL homolog, uncovers novel aspects of shoot meristem regulation and leaf blade development in Medicago truncatula. J Exp Bot 70:149–163. doi: 10.1093/jxb/ery346 30272208 PMC6305195

[76] Jha P, Ochatt SJ, Kumar V (2020) WUSCHEL: a master regulator in plant growth signaling. Plant Cell Rep 39:431–444. doi: 10.1007/s00299-020-02511-5 31984435

[77] Gordon SP, Chickarmane VS, Ohno C, Meyerowitz EM (2009) Multiple feedback loops through cytokinin signaling control stem cell number within the Arabidopsis shoot meristem. Proc Natl Acad Sci U S A 106:16529–16534. doi: 10.1073/pnas.0908122106 19717465 PMC2752578

[78] Chandler JW, Cole M, Flier A, Grewe B, Werr W (2007) The AP2 transcription factors DORNROSCHEN and DORNROSCHEN-LIKE redundantly control Arabidopsis embryo patterning via interaction with PHAVOLUTA. Development 134:1653–1662. doi: 10.1242/dev.001016 17376809

[79] Hirakawa Y, Kondo Y, Fukuda H (2010) TDIF peptide signaling regulates vascular stem cell proliferation via the WOX4 homeobox gene in Arabidopsis. Plant Cell 22:2618–2629. doi: 10.1105/tpc.110.076083 20729381 PMC2947162

[80] Han S, Cho H, Noh J, Qi J, Jung HJ, Nam H, et al (2018) BIL1-mediated MP phosphorylation integrates PXY and cytokinin signalling in secondary growth. Nat Plants 4:605–614. doi: 10.1038/s41477-018-0180-3 29988154

[81] Smit ME, McGregor SR, Sun H, et al (2020) A PXY-Mediated Transcriptional Network Integrates Signaling Mechanisms to Control Vascular Development in Arabidopsis. Plant Cell 32:319–335. doi: 10.1105/tpc.19.00562 31806676 PMC7008486

[82] Truernit E, Haseloff J (2007) A Role for KNAT Class II Genes in Root Development. Plant Signal Behav 2:10–12. doi: 10.4161/psb.2.1.3604 19704797 PMC2633887

[83] Cnops G, Wang X, Linstead P, Van Montagu M, Van Lijsebettens M, Dolan L (2000) Tornado1 and tornado2 are required for the specification of radial and circumferential pattern in the Arabidopsis root. Development 127:3385–3394. doi: 10.1242/dev.127.15.3385 10887093

[84] Chiu WH, Chandler J, Cnops G, Van Lijsebettens M, Werr W (2007) Mutations in the TORNADO2 gene affect cellular decisions in the peripheral zone of the shoot apical meristem of Arabidopsis thaliana. Plant Mol Biol 63:731–744. doi: 10.1007/s11103-006-9105-z 17351828

[85] Hassani SB, Trontin JF, Raschke J, Zoglauer K, Rupps A (2022) Constitutive Overexpression of a Conifer WOX2 Homolog Affects Somatic Embryo Development in Pinus pinaster and Promotes Somatic Embryogenesis and Organogenesis in Arabidopsis Seedlings. Front Plant Sci. doi: 10.3389/fpls.2022.838421 35360299 PMC8960953

[86] Zeng J, Ding Q, Fukuda H, He XQ (2016) Fertilization Independent Endosperm genes repress NbGH3.6 and regulate the auxin level during shoot development in Nicotiana benthamiana. J Exp Bot 67:2207–2217. doi: 10.1093/jxb/erw024 26873977 PMC4809283

[87] Mosquna A, Katz A, Decker EL, Rensing SA, Reski R, Ohad N (2009) Regulation of stem cell maintenance by the Polycomb protein FIE has been conserved during land plant evolution. Development 136:2433–2444. doi: 10.1242/dev.035048 19542356

[88] Garcia C, Furtado de Almeida AA, Costa M, Britto D, Valle R, Royaert S, et al (2019) Abnormalities in somatic embryogenesis caused by 2,4-D: an overview. Plant Cell, Tissue and Organ Culture (PCTOC) 2019 137:2 137:193–212.

[89] Valencia-Lozano E, Ibarra JE, Herrera-Ubaldo H, De Folter S, Cabrera-Ponce JL (2021) Osmotic stress-induced somatic embryo maturation of coffee Coffea arabica L., shoot and root apical meristems development and robustness. Sci Rep. doi: 10.1038/s41598-021-88834-z 33958620 PMC8102543

[90] Son O, Hur YS, Kim YK, et al (2010) ATHB12, an ABA-inducible homeodomain-leucine zipper (HD-Zip) protein of Arabidopsis, negatively regulates the growth of the inflorescence stem by decreasing the expression of a gibberellin 20-oxidase gene. Plant Cell Physiol 51:1537–1547. doi: 10.1093/pcp/pcq108 20668225

[91] Park J, Lee HJ, Cheon CI, Kim SH, Hur YS, Auh CK, et al (2011) The Arabidopsis thaliana homeobox gene ATHB12 is involved in symptom development caused by geminivirus infection. PLoS One. doi: 10.1371/journal.pone.0020054 21625602 PMC3097238

[92] Moulia B, Der Loughian C, Bastien R, et al (2011) Integrative Mechanobiology of Growth and Architectural Development in Changing Mechanical Environments. 269–302.

[93] Yang L, Liu S, Lin R (2020) The role of light in regulating seed dormancy and germination. J Integr Plant Biol 62:1310–1326. doi: 10.1111/jipb.13001 32729981

[94] Sung DY, Kaplan F, Guy CL (2001) Plant Hsp70 molecular chaperones: Protein structure, gene family, expression and function. Physiol Plant 113:443–451.

[95] Houben M, Van de Poel B (2019) 1-Aminocyclopropane-1-Carboxylic Acid Oxidase (ACO): The Enzyme That Makes the Plant Hormone Ethylene. Front Plant Sci. doi: 10.3389/fpls.2019.00695 31191592 PMC6549523

[96] Binder BM (2020) Ethylene signaling in plants. J Biol Chem 295:7710–7725. doi: 10.1074/jbc.REV120.010854 32332098 PMC7261785

[97] Horstman A, Bemer M, Boutilier K (2017) A transcriptional view on somatic embryogenesis. Regeneration (Oxf) 4:201–216. doi: 10.1002/reg2.91 29299323 PMC5743784

[98] Fambrini M, Usai G, Pugliesi C (2022) Induction of Somatic Embryogenesis in Plants: Different Players and Focus on WUSCHEL and WUS-RELATED HOMEOBOX (WOX) Transcription Factors. Int J Mol Sci. doi: 10.3390/ijms232415950 36555594 PMC9781121

[99] Chanvivattana Y, Bishopp A, Schubert D, Stock C, Moon YH, Sung ZR, et al (2004) Interaction of Polycomb-group proteins controlling flowering in Arabidopsis. Development 131:5263–5276. doi: 10.1242/dev.01400 15456723

[100] ZhangC, TangY, TangS, et al (2024) An inducible CRISPR activation tool for accelerating plant regeneration. Plant Commun 100823. doi: 10.1016/j.xplc.2024.100823 38243597

[101] Li Y, Williams B, Dickman M (2017) Arabidopsis B‐cell lymphoma2 (Bcl‐2)‐associated athanogene 7 (BAG 7)‐mediated heat tolerance requires translocation, sumoylation and binding to WRKY 29. New Phytologist 214:695–705. doi: 10.1111/nph.14388 28032645

