# Supplementary figures and images for: Editing of SlWRKY29 by CRISPR-activation promotes somatic embryogenesis in Solanum lycopersicum cv. Micro-Tom
Source: PLoS One. 2024 Apr 1;19(4):e0301169. doi: 10.1371/journal.pone.0301169 (PMC10984418; doi:10.1371/journal.pone.0301169)

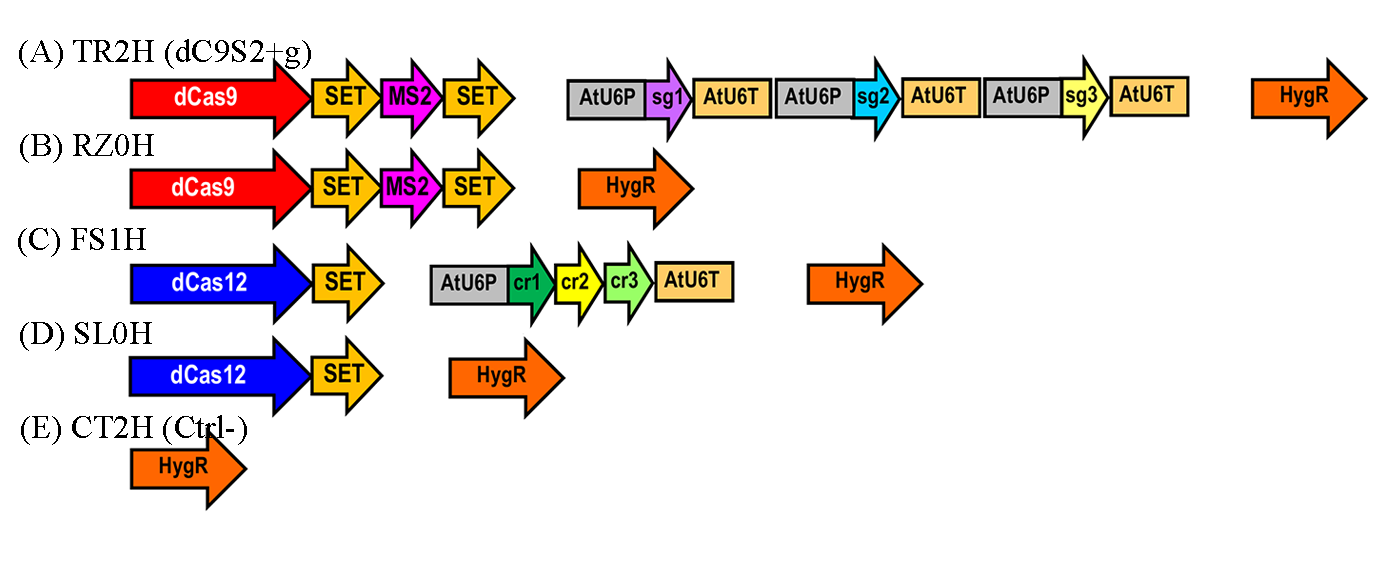

Supplement: S1 Fig — (A) dCas9S2+gRNA or TR2H vector, containing two SET domains fused each to dCas9 and MS2 protein. It also includes the sgRNA cassette containing three WRKY29 sgRNA’s each under the control of AtU6 promoter and AtU6 terminator. (B) RZ0H same as previous plasmids, but without expressing the sgRNA cassette (used as control). (C) FS1H, with a single SET domain fused to dCas12, and expressing an array of three WRKY29 crRNA’s units under the control of AtU6 promoter and AtU6 terminator. (D) SL0H, same as previous plasmid without expressing the respective crRNA cassette, as control. (E) CT2H, empty vector employed as control. All described plasmids contain a hygromycin resistance cassette as selection marker. For a detailed description of vector construction see S1 Table. (TIF) [file pone.0301169.s001.tif]

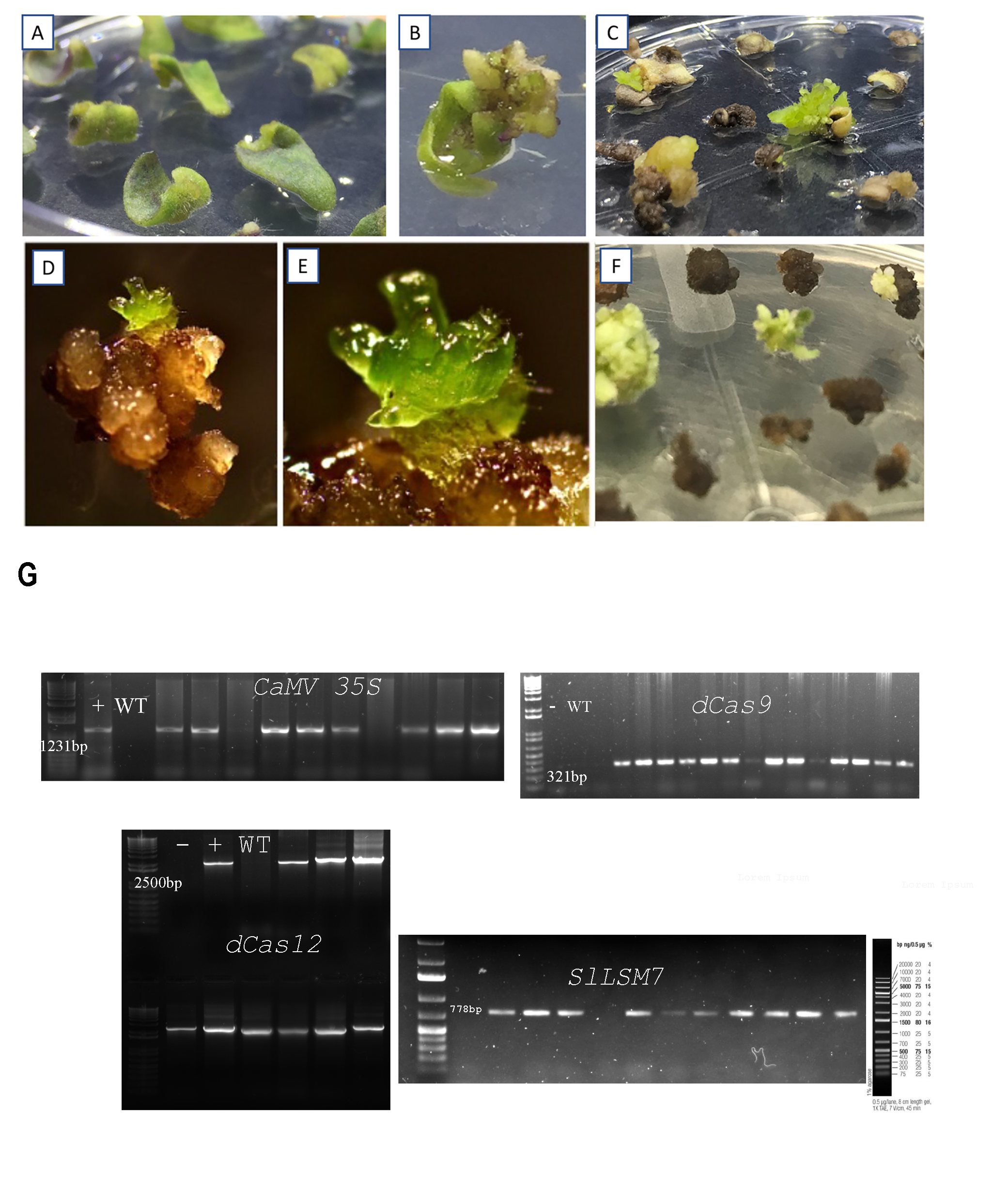

Supplement: S2 Fig — Individual transformation events sub-cultured onto fresh selective medium to evaluate the development of normal and anomalous structures. (A) Eight-days old cotyledons after biolistic treatment. (B) Induction of pro-embryogenic masses and development of somatic embryo-like structures growing on MS-BK2iP selective medium. (C) Primary selection on medium containing 9.6 mg/L of Hygromycin. (D) Isolation and selection of embryo-like events. (E) Close-up of (D). (F) Induction of secondary embryogenesis, for dissected and individually sub-cultured embryos onto fresh selective medium, to obtain embryogenic lines. (G) Genotyping of embryogenic lines. The presence of the transgene was confirmed by PCR with 35SCaMV, dCas9 and dCas12 specific primers. All embryogenic lines were genotyped (a few of the positive embryogenic lines are shown). Amplification of the SlLSM7 gene (endogenous gene) was used as control for the PCR reactions (for a list of primers used see S2 Table). (TIF) [file pone.0301169.s002.tif]

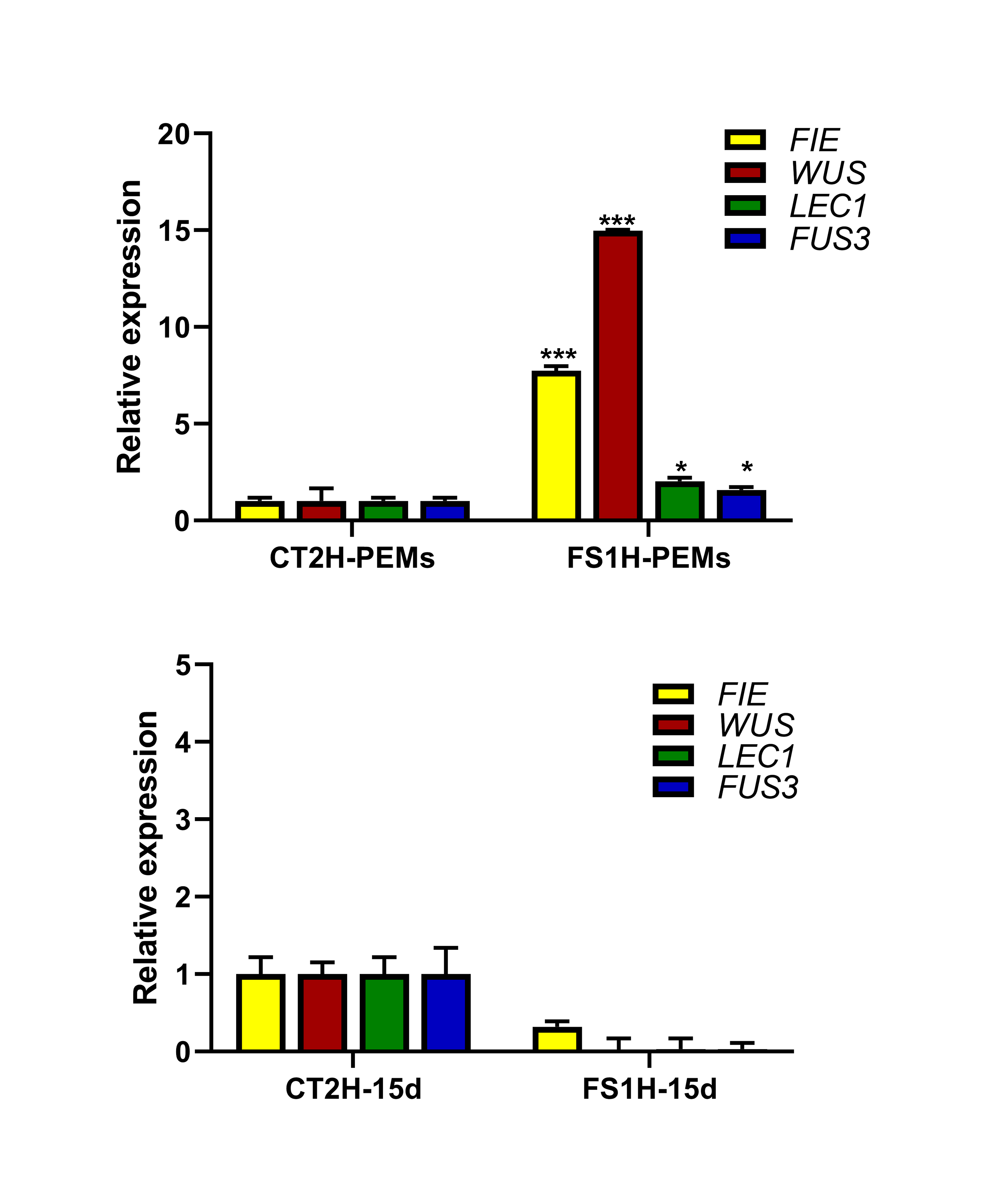

Supplement: S3 Fig — Samples were taken from pro-embryogenic masses (PEMs) and 15-days somatic embryos (in G9-2iP), and the relative expression was determined by qPCR. Data were normalized to the SlLSM7 reference gene (based on the 2−ΔΔCT method) [48]. Data represent mean ± SD from three independent experiments (n = 3). Statistical significance was determined with an unpaired two-tailed Student’s t-test (*p < 0.05, *** p < 0.001). (TIF) [file pone.0301169.s003.tif]

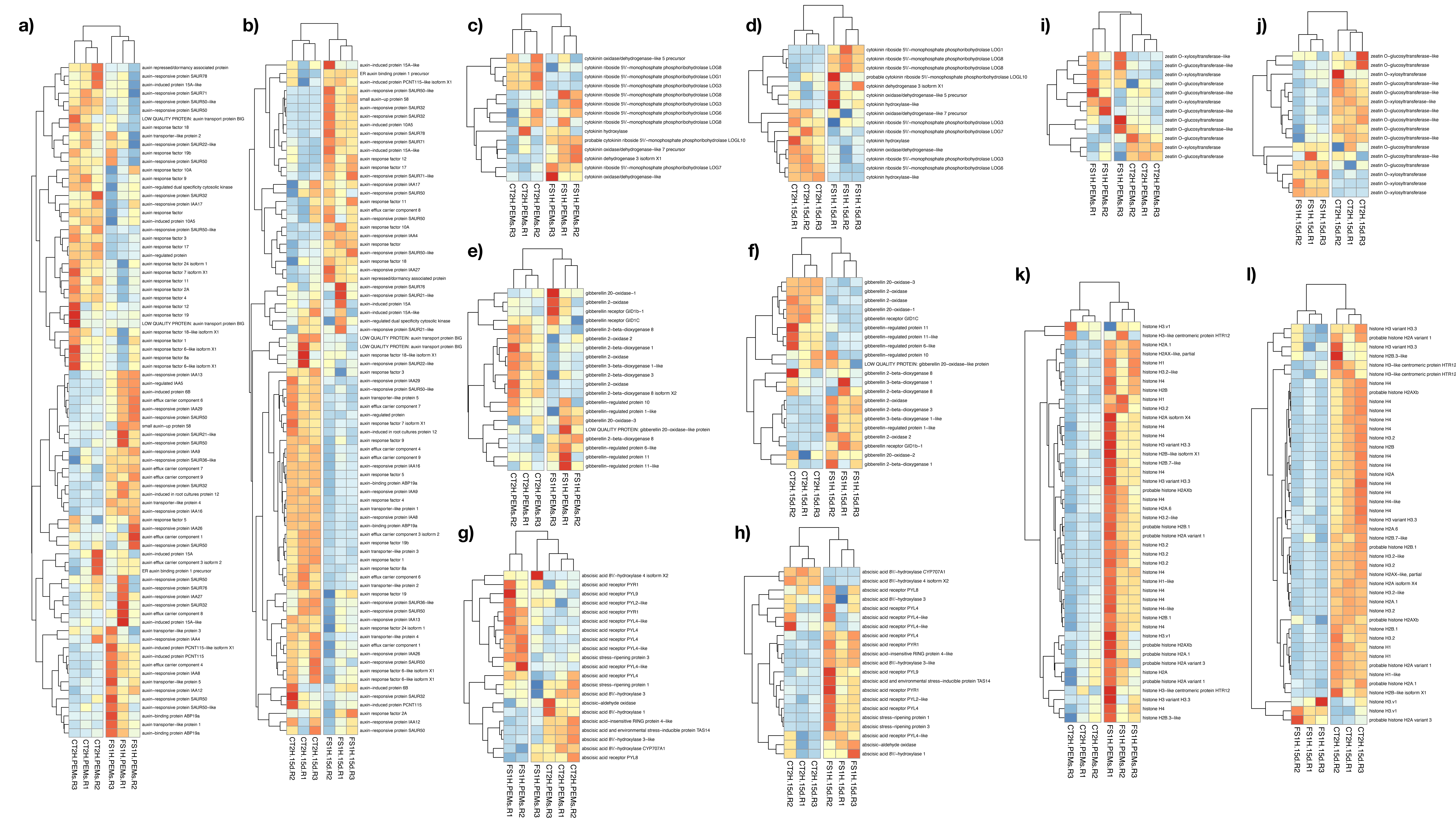

Supplement: S4 Fig — Heatmaps providing an overview on gene expression profiles, of the diverse pathways that were induced, based on the log2(fold-change) of ≥±1. Colors from yellow to red indicate up-regulation; colors from white to blue indicate down-regulation. (TIF) [file pone.0301169.s004.tif]

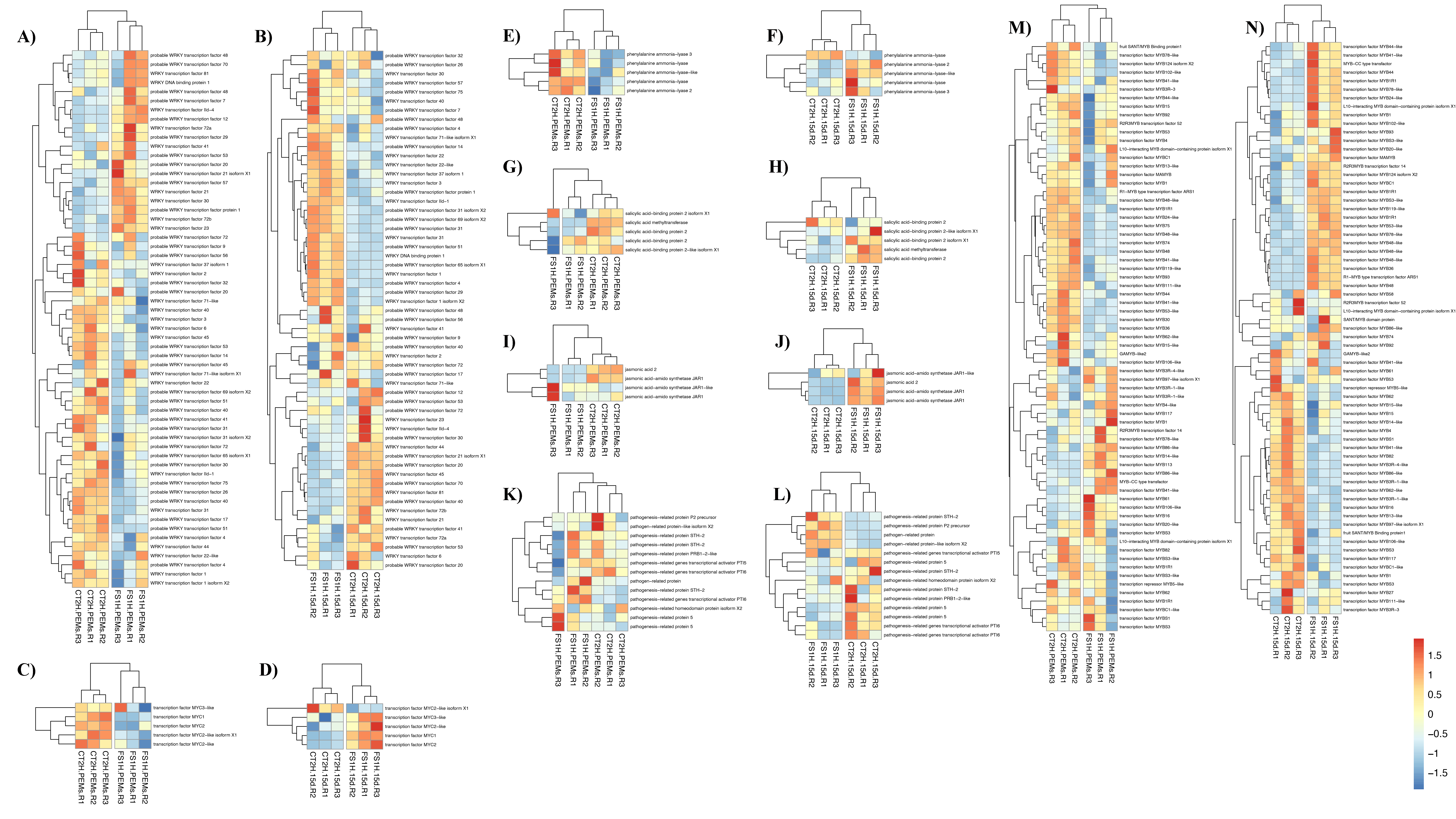

Supplement: S5 Fig — Heatmaps, providing an overview on gene expression profiles, of the diverse pathways that were induced, based on the log2(fold-change) of ≥±1. Colors from yellow to red indicate up-regulation; colors from white to blue indicate down-regulation. (TIF) [file pone.0301169.s005.tif]

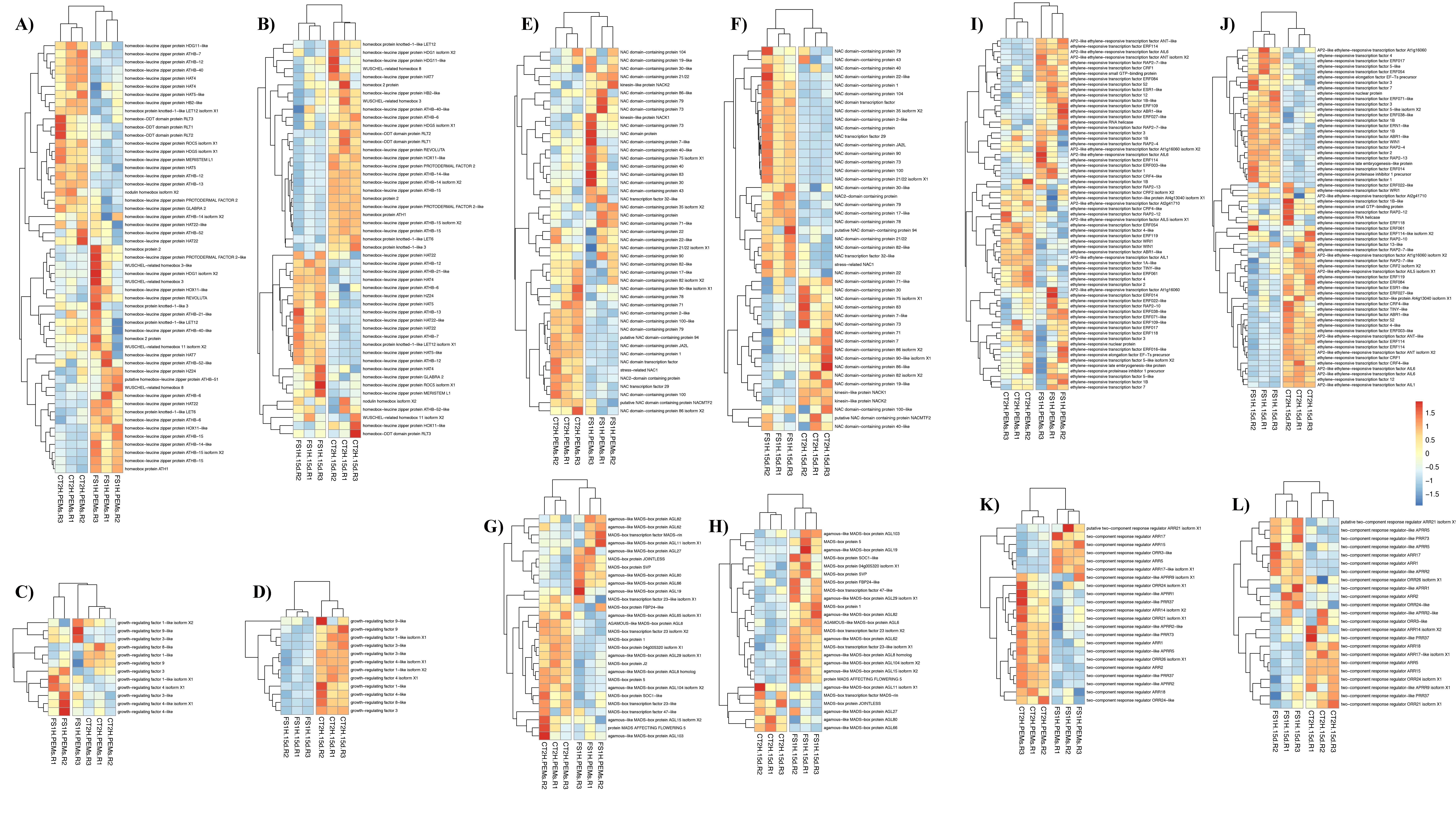

Supplement: S6 Fig — Heatmaps, providing an overview on gene expression profiles, of the diverse pathways that were induced, based on the log2(fold-change) of ≥±1. Colors from yellow to red indicate up-regulation; colors from white to blue indicate down-regulation. (TIF) [file pone.0301169.s006.tif]

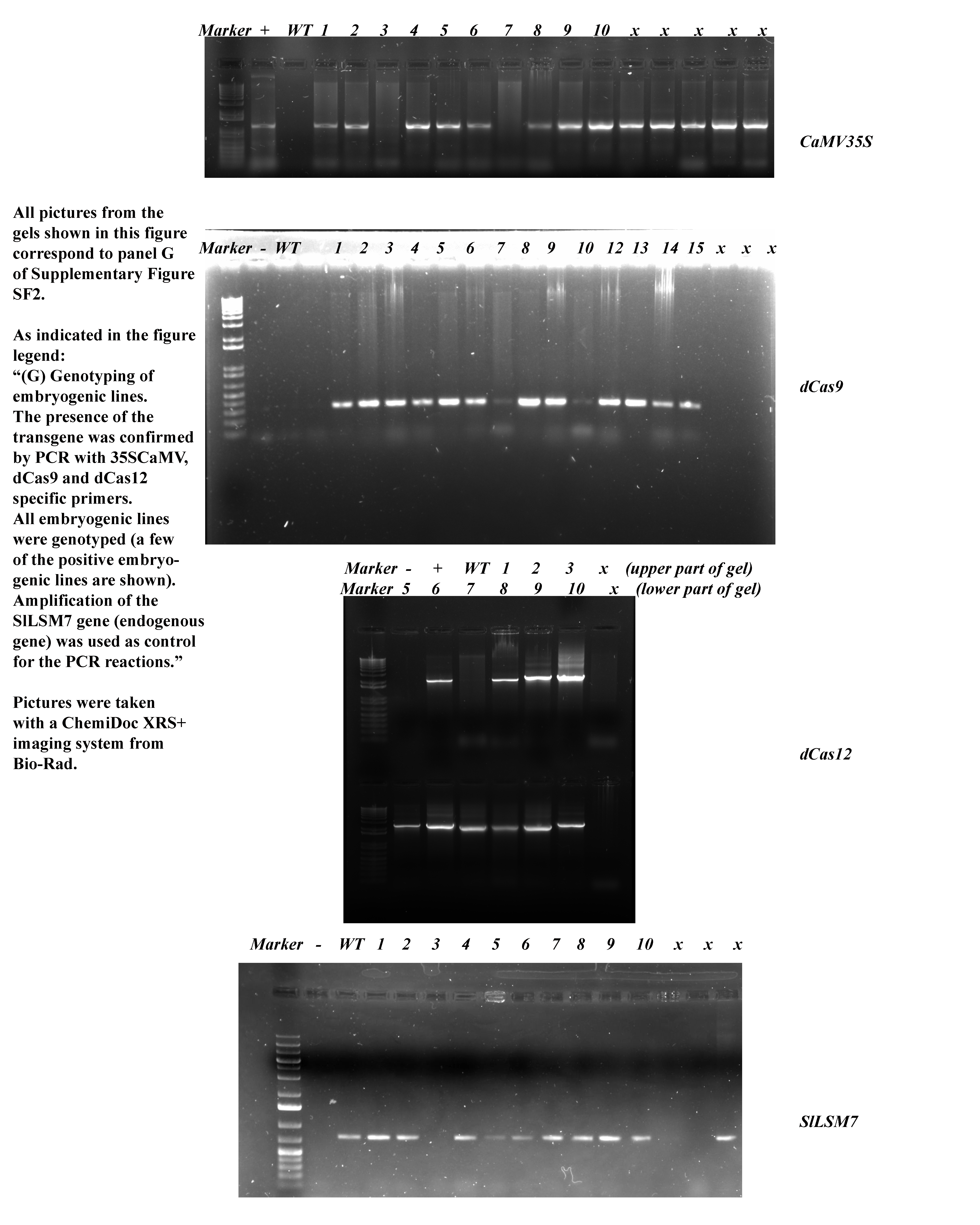

Supplement: S1 Raw images — (TIF) [file pone.0301169.s015.tif]
