# Supplementary material for: Editing of SlWRKY29 by CRISPR-activation promotes somatic embryogenesis in Solanum lycopersicum cv. Micro-Tom
Source: PLoS One. 2024 Apr 1;19(4):e0301169. doi: 10.1371/journal.pone.0301169 (PMC10984418; doi:10.1371/journal.pone.0301169)
Supplement: S1 Table — Vector construction. (DOCX) [file pone.0301169.s007.docx]

**S1 Table. Methods.** Vector construction

| 1. Preparation of the dCas9 entry vector with three gRNAs. The assembly method was based on both Golden Gate assembly and Gateway recombination [21]. First, for the dCas9 expression clone, the AtU6p promoter was PCR amplified (ST2-B Table) from the pICSL01009::AtU6p plasmid (Addgene.org, Plasmid #46968) and cloned separately at the XbaI-BglII sites of the pYPQ131A2.0 (Addgene #99884), pYPQ132A2.0 (Addgene #99887) and pYPQ133A2.0 (Addgene: #99891) vectors (substituting the original AtU6-1 promoter for the AtU6p consensus promoter sequence) [98]. The resulting plasmids were designated here as p131A2.0-AtU6p, p132A2.0-AtU6p and p133A2.0-AtU6p, respectively.   Next, the forward and reverse guide RNA (gRNA) oligonucleotides, targeting the promoter region of the *SlWRKY29* promoter region, were prepared for ligation (gRNA1, gRNA2, and gRNA3; see ST2-C Table). They were phosphorylated with the T4 polynucleotide kinase (Thermo Scientific, USA) [Lowder et al., 2017], and annealed with their corresponding pair by heating the reaction mix to 95^o^ C for 5 minutes and then by cooling the reaction slowly to room temperature (cooling ramp -5° C/min). The annealed oligos were ligated at the BsmBI sites of the p131A2.0-AtU6p, p132A2.0-AtU6p and p133A2.0-AtU6p plasmids, to produce the p131-AtU6p-sgWRKY29-1, p132A-AtU6p-sgWRKY29-2 and p133-AtU6p-sgWRKY29-3 vectors, correspondingly. Then, we performed a Golden Gate reaction to assemble the three gRNAs into the pYPQ143 plasmid (Addgene #69295), as recipient [Lowder et al., 2017]^a^. The resulting plasmid was designated as p143-3sg-WRKY29. |
| --- |
| 1. Assembly of the entry vector containing the CRISPR-Act2.0 module. For assembly of the CRISPR-Act2.0 system, plasmid pYPQ173 (Addgene #99907), which contains pco-dCas9-VP64 fusion protein and MS2-VP64 fusion protein linked by in-frame T2A sequence [Lowder et al., 2018], was used as the entry vector. In plasmid pYPQ173, the presence of the bacteriophage [coat protein](https://www.sciencedirect.com/topics/agricultural-and-biological-sciences/coat-protein) MS2 and its cognate RNA stem-loop aptamer scaffold help recruit additional transcriptional activators [Lowder et al., 2018]^b^. Furthermore, dCas9-VP64 and MS2-VP64 are linked by an in-frame T2A sequence, which encodes a self-cleaving 2A peptide (of 18 amino acids), allowing for production of two proteins from one mRNA via a translational skipping mechanism [23]. Concurrently, by making an MS2 fusion (pYPQ173) and expressing gRNAs in the gRNA2.0 scaffold, which contains MS2-binding aptamers, an increased number of transcriptional activators are recruited. Then, to generate the plasmid containing the SET-domain, as an epigenetic effector domain, the catalytic SET-domain from the *S. lycopersicum* histone lysine tri-methyltransferase *ATX1* gene ortholog (GeneID:100736465), was PCR amplified (see ST2-B Table) and cloned at the BamHI-AatII sites of the pYPQ173 plasmid (substituting the VP64 domain fused to MS2). The resultant entry plasmid was named p173-SET1. To substitute the VP64 domain fused to dCas9, in the p173-SET1 vector, a second PCR product containing the same SET-domain (see ST2-B Table for a list of oligos) was cloned at the SalI-AjiI sites, to obtain the entry plasmid p173SET12. |
| 1. Construction of the dCas12 entry vector with three crRNAs. We followed a strategy like the one developed by Wang and colleagues which takes into consideration that Cas12 is capable of processing pre-crRNA to release individual crRNAs without the need of an RNAse III [Wang et al., 2017]^c^. Thus, in our design, three units of crRNAs (crRNA array) in their mature form (20 bp LbCpf1 direct repeat DR sequences with 24 bp guide sequence), were ligated in tandem and driven by an AtU6 promoter in the same construct with LbCpf1 (Cas12 from *Lachnospiraceae bacterium*). The crRNA array was directly synthesized as a dsDNA fragment (Synbio Technologies, USA) and cloned at the BsaI sites of the p143-L2 plasmid to generate the entry vector p143-L2-crWRKY29. Beforehand, the p143-L2 plasmid was generated by replacing a SalI-PstI fragment from the p143-3sg-WRKY29 plasmid (as mentioned above) with a short 76 bp DNA fragment (or linker) to allow the cloning of the crRNA array. Sequences of the crRNA array and linker fragment are shown in ST2-D Table. |
| 1. Preparation of the entry vector containing the dCas12 module. A 612 bp fragment corresponding to the SET-domain from the *S. lycopersicum* *ATX1* gene ortholog was PCR amplified and cloned at the SalI-AatII sites of the pYPQ233 (Addgene # 86211), to produce the p233-SETX entry vector. |
| 1. Assembly of the destination vector. A 5905 bp PmeI-AflII fragment from the pK7WG2D.1 vector (<https://gatewayvectors.vib.be/collection/pk7wg2d>) was cloned at the PmeI-AflII sites of pYPQ203 (Addgene #86207) to generate the destination vector p203-GFP-Hyg. |
| 1. Generation of the empty vector. A 2628 bp SalI fragment was removed from the destination vector p203-GFP-Hyg, which was then re-circularized with the T4 ligase (Thermo Scientific, USA) to create the pEGFPHS empty vector. |
| 1. Generation of CRISPR-Act2.0 and CRISPR-dCas12 vectors via recombination. To generate the final expression vectors (Supplementary Figure SF1), we performed distinct recombination reactions combinations between the destination and entry vectors (as shown in ST3 Table), with the help of the Gateway LR clonase II Enzyme mix (Invitrogen, USA; following the manufacturer’s instructions). The pYPQ143 empty vector was used as entry vector to generate control plasmids without gRNA’s expression. The list of the expression vectors used to transform tomato explants is shown in SF1 Figure and ST3 Table. |

^a^Lowder, L.G., Paul, J.W. 3rd, Qi, Y. (2017). Multiplexed transcriptional activation or repression in plants using CRISPR-dCas9-based systems. In Kaufmann, K., Mueller-Roeber, B. (eds) Plant Gene Regulatory Networks. *Methods in Molecular Biology*, vol 1629. Humana Press, New York, NY. https://doi.org/10.1007/978-1-4939-7125-1_12

^b^Lowder, L.G., Zhou, J., Zhang, Y., Malzahn, A., Zhong, Z., Hsieh, T.F., et al. (2018). Robust transcriptional activation in plants using multiplexed CRISPR-Act2.0 and mTALE-act systems. *Mol Plant.* 11, 245-256. doi: 10.1016/j.molp.2017.11.010.

^c^Wang, M., Mao, Y., Lu, Y., Tao, X., Zhu, J.K. (2017). Multiplex Gene Editing in Rice Using the CRISPR-Cpf1 System. *Mol Plant*. 10, 1011-1013. doi: 10.1016/j.molp.2017.03.001.
