# Supplementary material for: Editing of SlWRKY29 by CRISPR-activation promotes somatic embryogenesis in Solanum lycopersicum cv. Micro-Tom
Source: PLoS One. 2024 Apr 1;19(4):e0301169. doi: 10.1371/journal.pone.0301169 (PMC10984418; doi:10.1371/journal.pone.0301169)
Supplement: S2 Table — (A) Selected target sequences at the SlWRKY29 promoter region for dCas9 and dCas12. Highlighted sequences correspond to the PAM sequence. (B) Oligonucleotides used for vector construction. (C) SlWRKY29 gRNA oligonucleotide cloning reference. (D) Linker sequence in the p143-L2 plasmid and synthetic DNA with the crRNAs (crRNA array) for dCas12. (E) List of oligos used for qPCR. (F) List of oligos used for PCR-genotyping. (DOCX) [file pone.0301169.s008.docx]

**S2 Table. DNA sequences**.

| Nuclease | gRNA | Target | Position of target sequences at the tomato genome |
| --- | --- | --- | --- |
| dCas9 | sgWRKY29-1 | AGCTAAAAATATGAAGTTTGAG**TGG** | SL3.0ch08:-64728574 |
|  | sgWRKY29-2 | CAAAGTCAAAGGGTAACGACAG**CGG** | SL3.0ch08:-64728436 |
|  | sgWRKY29-3 | TGAAAAAATCATTTAAGTACGT**AGG** | SL3.0ch08:+64728327 |
| dCas12 | WRKY29_crRNA1 | **TTTA**GCTATCAAAAAAGTCAGCGCCATT | SL3.0ch08:+64728568 |
|  | WRKY29_crRNA2 | **TTTG**AGTGGTATTTAAAGACTAAAAATA | SL3.0ch08:-64728579 |
|  | WRKY29_crRNA3 | **TTTA**CCCTAATTAGCTTCCGCTGTCGTT | SL3.0ch08:+64728395 |

(A) Selected target sequences at the *SlWRKY29* promoter region for dCas9 and dCas12. Highlighted sequences correspond to the PAM sequence.

| Name | Sequence 5’ – 3’ |
| --- | --- |
| pU6-131-Fw | TCCTCTAGAGGTCTCGCTATTGATCAAAAGTCCCACATCG |
| pU6-132-Fw | TCCTCTAGAGGTCTCCCATGTGATCAAAAGTCCCACATCG |
| pU6-133-Fw | TCCTCTAGAGGTCTCAGGACTGATCAAAAGTCCCACATCG |
| pU6-A2.0-Rv | ACTAGATCTCGTCTCCAATCGCTATGTCGACTCTATC |
| SET-BamHI-Fw | TAAGCAGGATCCATGGTTGAGAAATACAACTACATGA |
| SET-SalI-Fw | TAAGCAGTCGACATGGTTGAGAAATACAACTACATGA |
| SET-AatII-Rv | TGCTTAGACGTCTCAGTAAGCAAGCAACCAAAGGCACCTC |
| SET-T2A-Rv | GTCACCGCATGTTAGAAGACTTCCTCTGCCCTCTTCTCCTTCCCAATCTATTAACTCACTGC |
| Link2-F | TCGACATAGCGATTGAATTTCTACTAAGTGTAGATTGAGACCGTTAACTGACCATGGACGGTCTCAAGCGTCTGCA |
| Link2-R | GACGCTTGAGACCGTCCATGGTCAGTTAACGGTCTCAATCTACACTTAGTAGAAATTCAATCGCTATG |

(B) Oligonucleotides used for vector construction.

| Oligo name | gRNA oligo sequence with overhangs in bold for annealing and subcloning. In lower case, guanine recognized by RNA pol III required for transcription directed by the U6 promoter | Direction on promoter | gRNA entry plasmid cloning correspondence |
| --- | --- | --- | --- |
| sgWRKY29-1-F | **GATT**gAGCTAAAAATATGAAGTTTGAG | Reverse | p131-AtU6p |
| sgWRKY29-1-R | **AAAC**CTCAAACTTCATATTTTTAGCTc |  |  |
| sgWRKY29-2-F | **GATT**gCAAAGTCAAAGGGTAACGACAG | Reverse | P132-AtU6p |
| sgWRKY29-2-R | **AAAC**CTGTCGTTACCCTTTGACTTTGc |  |  |
| sgWRKY29-3-F | **GATT**gTGAAAAAATCATTTAAGTACGT | Forward | P133-AtU6p |
| sgWRKY29-3-R | **AAAC**ACGTACTTAAATGATTTTTTCAc |  |  |

(C) *SlWRKY29* gRNA oligonucleotide cloning reference.

| Name | Sequence 5’ – 3’ | Description |
| --- | --- | --- |
| Linker L2 | CGACATAGCGATTGAATTTCTACTAAGTGTAGATT**GAGACC**GTTAACTGACCATGGAC**GGTCTC**AAGCGTC*TGCA* | *Sal*I-DR-***Bsa*I**-***Bsa*I**-*PstI* |
| cr-Array | **GGTCTC**AAGATGCTATCAAAAAAGTCAGCGCCATTAATTTCTACTAAGTGTAGATAGTGGTATTTAAAGACTAAAAATAAATTTCTACTAAGTGTAGATCCCTAATTAGCTTCCGCTGTCGTTAATTTCTACTAAGTGTAGATTTTTTTTCTCGAGAGCGT**GAGACC** | ***Bsa*I**-WRKY29_crRNA1-DR-WRKY29_crRNA2-DR- WRKY29_crRNA3-DR-Poly T-***Bsa*I** |

(D) Linker sequence in the p143-L2 plasmid and synthetic DNA with the crRNAs (crRNA array) for dCas12.

| **Gene** | Name, NCBI, SOL GENOMICS NETWORK | Primer 5´-3´ | Amplicon (bp) |
| --- | --- | --- | --- |
| *SlWRKY29* | GeneID: 101245784, XM_004245516.4 | F: ctccttatccaaggagttattataggtg | 156 |
|  |  | R: cagctagagaatttctacgtgttggctg |  |
| *SlWUS* | NM_001247086.3 | F: ccgcctctgccactgataat | 124 |
|  |  | R: gatggacactggacacctgg |  |
| *SlFIE* | NM_001247555.2 | F: tctctaagctctggcggagt | 132 |
|  |  | R: gcgtttgccttcttggagtc |  |
| *SlLEC1-like* | Solyc05g005370.1.1 | F: ctctgatgttgttgtgtggaac | 144 |
|  |  | R: atcctgcgcatgattttgacca |  |
| *SlFUS3* | XM_004231700.4 | F: tgatgaatcctccaactcccac | 141 |
|  |  | R: ttggttcaagtacacgtgcg |  |
| *SlLSM7* | U6 snRNA-associated Sm-like protein LSm7,  Solyc09g009640; K4CQZ3 | F: gtggaagacaagtggttggaacac | 220 |
|  |  | R: cgtctggctgaacaaaaggattgg |  |
| *SlTIP41* | TIP41-interacting protein; Solyc10g049850.3 | F: atggagtttttgagtcttctgc | 235 |
|  |  | R: gctgcgtttctggcttagg |  |

(E) List of oligos used for qPCR.

| **Gene/**  **Sequence** | Name, NCBI,  SOL GENOMICS NETWORK | Primer 5´-3´ | Amplicon (bp) |
| --- | --- | --- | --- |
| *dCas12* | Addgene pYPQ233 plasmid sequence: (Addgene plasmid # 86211 ; http://n2t.net/addgene:86211 ; RRID:Addgene_86211) (primers amplify a fragment from the dCas12a to the attR5 sequence) | F-dCas12: ttatataccggcgtggcttac | 2453 |
|  |  | R-ATTR5: accactttgtacaagaaagctg |  |
| *dCas9* | Addgene pYPQ173 plasmid sequence (Addgene plasmid # 99907 ; http://n2t.net/addgene:99907 ; RRID:Addgene_99907) (primers amplify a fragment of the pco-dCas9) | F: gttgttgataagggagcttc | 321 |
|  |  | R: gaatctatcctcaactccag |  |
| *35SCaMV* | Addgene pYPQ203 plasmid sequence (Addgene plasmid # 86207 ; http://n2t.net/addgene:86207 ; RRID:Addgene_86207) (primers amplify a fragment from the 35SCaMV promoter region to the CaMV poly-A signal; includes the HygR sequence) | 35S Prom F: tccttcgcaagacccttc | 1231 |
|  |  | 35S Term R: ccttatctgggaactactcacac |  |
| *SlLSM7*  (endogenous gene) | U6 snRNA-associated Sm-like protein LSm7,  Solyc09g009640; K4CQZ3 | F: gtggaagacaagtggttggaacac | 778 |
|  |  | R: cgtctggctgaacaaaaggattgg |  |

(F) List of oligos used for PCR-genotyping.
