# Supplementary material for: Editing of SlWRKY29 by CRISPR-activation promotes somatic embryogenesis in Solanum lycopersicum cv. Micro-Tom
Source: PLoS One. 2024 Apr 1;19(4):e0301169. doi: 10.1371/journal.pone.0301169 (PMC10984418; doi:10.1371/journal.pone.0301169)
Supplement: S3 Table — Recombination reactions between destination and entry vectors to generate the CRISPR-Act2.0 and CRISPR-dCas12 expression vectors used to transform tomato explants via biolistics. (DOCX) [file pone.0301169.s009.docx]

**S3 Table. Recombination reactions.** Recombination reactions between destination and entry vectors to generate the CRISPR-Act2.0 and CRISPR-dCas12 expression vectors used to transform tomato explants via biolistics.

| Key | Plasmid | p203-GFP-Hyg | pYPQ173 | p173-SET1 | p173-SET12 | p143-3sg-WRKY29 | pYPQ143 | p233-SETX | P143-L2-crRNA's | Expression vectors/Description |
| --- | --- | --- | --- | --- | --- | --- | --- | --- | --- | --- |
| TR2H = | pCRISPR2.0-SET12-RNA's | + |  |  | + | + |  |  |  | dCas9:SET/MS2:SET/sgRNA’s |
| RZ0H = | pCRISPR2.0-SET12-_Δ_RNA's | + |  |  | + |  | + |  |  | dCas9:SET/MS2:SET |
| FS1H = | pdLbCpf1-SET-crRNA's | + |  |  |  |  |  | + | + | dCas12:SET/crRNA’s |
| SL0H = | pdLbCpf1-SET-_Δ_crRNA's | + |  |  |  |  | + | + |  | dCas12:SET |
| CT2H = | pEGFPHS |  |  |  |  |  |  |  |  | Empty plasmid |
