# Supplementary material for: Editing of SlWRKY29 by CRISPR-activation promotes somatic embryogenesis in Solanum lycopersicum cv. Micro-Tom
Source: PLoS One. 2024 Apr 1;19(4):e0301169. doi: 10.1371/journal.pone.0301169 (PMC10984418; doi:10.1371/journal.pone.0301169)
Supplement: S4 Table — (A) Osmotic treatment medium. (B) Conventional medium. (C) Selective medium. (D) Maturation, rooting and elongation medium. (DOCX) [file pone.0301169.s010.docx]

**S4 Table. Media.**

| MS-BK2iP medium  (high osmotic medium) | MS basal medium (Sigma-Aldrich cat. # M5519; according to Murashige and Skoog, 1962), supplemented with 1 mg/L 2iP (N^6^-(Δ^2^-isopentenyladenine), 1 mg/L kinetin, 2 mg/L BAP, 5 g/L gelrite, 50 g/L sucrose, pH 5.8 |
| --- | --- |
| MS-NAA/BAP medium (conventional osmotic medium) | MS basal medium (Sigma-Aldrich cat. # M5519; according to Murashige and Skoog, 1962), supplemented with 0.1 mg/L of NAA (naphthaleneacetic acid), 100 mg/L de thiamin, 8 g/L agar, 30 g/L sucrose, pH 5.8 (modified from Pino et al., 2010)^a^ |
| MS-Zea medium  (high osmotic medium) | MS basal medium (Sigma-Aldrich cat. # M5519; according to Murashige and Skoog, 1962)^b^, supplemented with 1.5 mg/L trans-Zeatin (Caisson Labs, USA, cat. #1637-39-4), 8 g/L agar, 5 g/L gelrite, 50 g/L sucrose, pH 5.8 |

(A) Osmotic treatment medium.

| MS-BK2iP medium | MS basal medium supplemented with 1 mg/L 2iP (N^6^-(Δ^2^-isopentenyladenine), 1 mg/L kinetin, 2 mg/L BAP, 3 g/L gelrite, 30 g/L sucrose, pH 5.8 |
| --- | --- |
| MS-NAA/BAP medium | MS basal medium supplemented with 1.0 mg/L of BAP (6-Benzyladenine; Sigma-Aldrich cat. #B3274) 6 g/L agar, 30 g/L sucrose, pH 5.8 (modified from Pino et al., 2010) |
| MS-Zea medium | MS basal medium supplemented with 1.5 mg/L trans-Zeatin (Caisson Labs, USA, cat. #1637-39-4), 3 g/L gelrite, 30 g/L sucrose, pH 5.8 |

(B) Conventional medium.

| MS- BK2iP medium | MS basal medium supplemented with 1 mg/L 2iP (N^6^-(Δ^2^-isopentenyladenine), 1 mg/L kinetin, 2 mg/L BAP, 3 g/L gelrite, 30 g/L sucrose and 9.5 mg/L of hygromycin, pH 5.8 |
| --- | --- |
| MS-NAA/BAP medium | MS basal medium supplemented with 1.0 mg/L of BAP (6-Benzyladenine; Sigma-Aldrich cat. #B3274) 6 g/L agar, 30 g/L sucrose, and 9.5 mg/L of hygromycin, pH 5.8 (modified from Pino et al., 2010) |
| MS-Zea medium | MS basal medium supplemented with 1.5 mg/L trans-Zeatin (Caisson Labs, USA, cat. #1637-39-4) 3 g/L gelrite, 30 g/L sucrose and 9.5 mg/L of hygromycin, pH 5.8 |

(C) Selective medium.

| G9-2iP medium | MS basal medium supplemented with 0.2 mg/L of 2iP (N^6^-(Δ^2^-isopentenyladenine), 9 g/L gelrite and 10 g/L glucose (filter-sterilized), pH 5.8 |
| --- | --- |
| G9-Zea medium | MS basal medium supplemented with 0.2 mg/L trans-Zeatin (Caisson Labs, USA, cat. #1637-39-4) 9 g/L gelrite, 10 g/L glucose (filter-sterilized) and pH 5.8 |

(D) Maturation, rooting and elongation medium.

^a^Pino, L.E., Lombardi-Crestana, S., Azevedo, M.S., Scotton, D.C., Borgo, L., Quecini, V., et al. (2010). The Rg1 allele as a valuable tool for genetic transformation of the tomato 'Micro-Tom' model system. *Plant Methods* 6, 1-11. <https://doi.org/10.1186/1746-4811-6-23>

^b^Murashige T, Skoog F. A. (1962). Revised medium for rapid growth and bio assays with tobacco tissue cultures. *Physiol Plant* 15, 473–97. <https://onlinelibrary.wiley.com/doi/full/10.1111/j.1399-3054.1962.tb08052.x>
