# Supplementary material for: Editing of SlWRKY29 by CRISPR-activation promotes somatic embryogenesis in Solanum lycopersicum cv. Micro-Tom
Source: PLoS One. 2024 Apr 1;19(4):e0301169. doi: 10.1371/journal.pone.0301169 (PMC10984418; doi:10.1371/journal.pone.0301169)
Supplement: S5 Table — (A) Sample sequencing statistics of RNA-seq libraries of S. lycopersicum transcriptome. (B) Pairwise comparisons. (DOCX) [file pone.0301169.s011.docx]

**S5 Table. RNA-seq libraries.**

| **Sample ID (treatment)** | **Barcode Sequence** | **# Reads** | **Yield (Mbases)** | **Mean Quality Score** | **% Bases >= 30** |
| --- | --- | --- | --- | --- | --- |
| FS1H-PEMs-R1 | TCCGCGAA+GCAAGCAA | 39943423 | 11983 | 35.96 | 93.85 |
| FS1H-PEMs-R2 | TCCGCGAA+AACCTCTC | 38927522 | 11678 | 35.97 | 93.87 |
| FS1H-PEMs-R3 | TCCGCGAA+AACCAGGT | 36829751 | 11049 | 35.92 | 93.63 |
| CT2H-PEMs-R1 | CGGCTATG+AACCAGGT | 37070527 | 11121 | 35.86 | 93.34 |
| CT2H-PEMs-R2 | CGGCTATG+TTCCGCTT | 36992595 | 11098 | 35.88 | 93.42 |
| CT2H-PEMs-R3 | CGGCTATG+TTGTTCCG | 39725422 | 11918 | 35.8 | 92.97 |
| FS1H-15d-R1 | TCCGCGAA+TTCCGCTT | 42277789 | 12683 | 35.92 | 93.6 |
| FS1H-15d-R2 | TCCGCGAA+TTGTTCCG | 40546875 | 12164 | 35.95 | 93.77 |
| FS1H-15d-R3 | TCCGCGAA+AGCTTACC | 40043138 | 12013 | 35.88 | 93.37 |
| CT2H-15d-R1 | CGGCTATG+AGCTTACC | 38980816 | 11694 | 35.87 | 93.35 |
| CT2H-15d-R2 | CGGCTATG+ATGCCACA | 34710262 | 10413 | 35.92 | 93.67 |
| CT2H-15d-R3 | CGGCTATG+TCCGTAGT | 39423301 | 11827 | 35.86 | 93.3 |

(A) Sample sequencing statistics of RNA-seq libraries of *S. lycopersicum* transcriptome.

- Control CT2H-PEMs-R1, CT2H-PEMs-R2, CT2H-PEMs-R3 -vs- FS1H-PEMs-R1, FS1H-PEMs-R2, FS1H-PEMs-R3, (dCas12:SET + crRNA) before induction
- Control CT2H-15d-R1, CT2H-15d-R2, CT2H-15d-R3 -vs- FS1H-15d-R1, FS1H-15d-R2, FS1H-15d-R3, (dCas12:SET + crRNA) after induction

(B) Pairwise comparisons.
