# Supplementary material for: Editing of SlWRKY29 by CRISPR-activation promotes somatic embryogenesis in Solanum lycopersicum cv. Micro-Tom
Source: PLoS One. 2024 Apr 1;19(4):e0301169. doi: 10.1371/journal.pone.0301169 (PMC10984418; doi:10.1371/journal.pone.0301169)
Supplement: S6 Table — (DOCX) [file pone.0301169.s012.docx]

**S6 Table. List of transcripts that were exclusively detected in the FS1H samples (PEMs and 15-days embryos in G9-2iP).**

| ***S. lycopersicum* accesion number** | ***A. thaliana* ortholog** | **Annotation - PEMs** |
| --- | --- | --- |
| **Solyc06g076000.1.1** | WOX2 | annotation not available |
| **Solyc02g078190.2.1** | VATG3 | annotation not available |
| **Solyc03g118560.1.1** | TDX | annotation not available |
| **Solyc10g084910.1.1** | TAC1 | annotation not available |
| **Solyc03g093770.1.1** | SUVH6 | annotation not available |
| **rpl22** | RPL22 | 30S ribosomal protein S3, chloroplastic ; Belongs to the universal ribosomal protein uS3 family |
| **rpl20** | RPL20 | 50S ribosomal protein L20, chloroplastic; Binds directly to 23S ribosomal RNA and is necessary for the in vitro assembly process of the 50S ribosomal subunit. It is not involved in the protein synthesizing functions of that subunit |
| **Solyc08g078200.1.1** | PPa5 | annotation not available |
| **Solyc03g096040.2.1** | PER1 | Uncharacterized protein; 1-cysteine peroxiredoxin 1 |
| **Solyc09g074760.1.1** | NF--YB5 | annotation not available |
| **Solyc07g052300.2.1** | MYB101 | Plasma membrane atpase 1-like; Plasma membrane ATPase; H(+)-ATPase 7 |
| **Solyc11g011560.1.1** | MMD1 | annotation not available |
| **Solyc12g013940.1.1** | LUG | annotation not available |
| **Solyc05g005370.1.1** | LEC1 | annotation not available |
| **Solyc03g044440.1.1** | L22p/L17e | annotation not available |
| **Solyc07g065560.1.1** | HBT11 | annotation not available |
| **Solyc08g013680.2.1** | HA11 | annotation not available |
| **Solyc02g094460.1.1** | FUS3 | annotation not available |
| **FIE** | FIE | Fertilization-independent endosperm protein |
| **Solyc08g074860.1.1** | EIF3G1 | Uncharacterized protein loc104646564; Belongs to the thioredoxin family |
| **Cyclin-D5-3-like** | **Cyclin-D5-3-like** | B3 domain-containing transcription factor FUS3; Uncharacterized protein; AP2/B3-like transcriptional factor family protein |
| **Solyc05g052080.1.1** | AT5G07490 | Wuschel-related homeobox 2; Uncharacterized protein; WUSCHEL related homeobox 2 |
| **Solyc06g005970.2.1** | AT3G47000 | Uncharacterized protein; Belongs to the cyclin family |
| **Solyc01g090830.2.1** | AT1G02040 | V-type proton ATPase subunit G; Catalytic subunit of the peripheral V1 complex of vacuolar ATPase (V-ATPase). V-ATPase is responsible for acidifying a variety of intracellular compartments in eukaryotic cells |
| **Solyc09g060000.1.1** | ASHH1 | annotation not available |
| **Solyc10g005680.1.1** | WRKY2 | WRKY2 |

| ***S. lycopersicum* accesion number** | ***A. thaliana* ortholog** | **STRING** | **Annotation – 15-days embrios (G9)** |
| --- | --- | --- | --- |
| **SlZFP2** | ZFP2 | Solyc07g006880.1.1 | zinc finger transcription factor SlZFP2 |
| **Solyc02g078190.2.1** | VATG3 | Solyc02g078190.2.1 | V-type proton ATPase subunit G 1-like |
| **Solyc01g088010.1.1** | GLYI8 | Solyc01g088010.1.1 | uncharacterized protein LOC101261159 |
| **Solyc05g041140.2.1** | AT3G53980 | Solyc05g041140.2.1 | uncharacterized protein LOC101257668 |
| **Solyc08g006800.1.1** | AT4G31830 | Solyc08g006800.1.1 | uncharacterized protein LOC101251603 |
| **Solyc09g089590.1.1** | SUP | Solyc09g089590.1.1 | transcriptional regulator SUPERMAN-like |
| **Solyc06g082450.1.1** | OFP17 | Solyc06g082450.1.1 | transcription repressor OFP17 |
| **Solyc03g112890.1.1** | MYB98 | Solyc03g112890.1.1 | transcription factor MYB98-like |
| **Solyc02g082380.1.1** | AT1G49010 | Solyc02g082380.1.1 | transcription factor MYB1R1-like |
| **Solyc12g049350.1.1** | MYB12 | Solyc12g049350.1.1 | transcription factor MYB12-like isoform X2 |
| **Solyc08g013680.2.1** | PPA5 | Solyc08g013680.2.1 | soluble inorganic pyrophosphatase PPA1-like |
| **NRAMP1** | NRAMP1 | Solyc11g018530.1.1 | root-specific metal transporter |
| **Solyc11g013320.1.1** | KOM | Solyc11g013320.1.1 | RHOMBOID-like protein 8 |
| **Solyc08g014310.2.1** | SPDS2 | Solyc08g014310.2.1 | putrescine N-methyltransferase 2 |
| **pmt** | SPDS1 | Solyc06g053510.2.1 | putrescine N-methyltransferase |
| **Solyc04g074320.1.1** | TT1 | Solyc04g074320.1.1 | protein TRANSPARENT TESTA 1 |
| **SP3D** | FT | Solyc03g063100.1.1 | protein single flower truss |
| **SP6A** | FT | Solyc05g055660.1.1 | protein SELF PRUNING 6A |
| **SP5G** | FT | Solyc05g053850.2.1 | protein SELF PRUNING 5G |
| **Solyc03g120250.2.1** | SULTR3;4 | Solyc03g120250.2.1 | probable sulfate transporter 3.4 |
| **Solyc01g087460.1.1** | AT2G34740 | Solyc01g087460.1.1 | probable protein phosphatase 2C 58 |
| **Solyc03g059080.1.1** | AT2G41970 | Solyc03g059080.1.1 | probable protein kinase At2g41970 isoform X2 |
| **Solyc08g078200.1.1** | HA11 | Solyc08g078200.1.1 | plasma membrane ATPase 1-like |
| **Solyc02g082090.2.1** | AT1G30870 | Solyc02g082090.2.1 | peroxidase 7 |
| **Solyc05g006230.1.1** | AT1G24110 | Solyc05g006230.1.1 | peroxidase 6 |
| **Solyc12g087830.1.1** | MAF3 | Solyc12g087830.1.1 | MADS-box transcription factor 23 |
| **Solyc10g017630.1.1** | AGL20 | Solyc10g017630.1.1 | MADS-box protein SOC1-like isoform X1 |
| **LOXB** | LOX1 | Solyc01g099190.2.1 | linoleate 9S-lipoxygenase B |
| **Solyc09g082110.2.1** | AT3G22490 | Solyc09g082110.2.1 | late embryogenesis abundant protein D-34-like |
| **Solyc09g091040.2.1** | UNE15 | Solyc09g091040.2.1 | late embryogenesis abundant protein 1 |
| **Solyc04g080660.2.1** | JMT | Solyc04g080660.2.1 | jasmonate O-methyltransferase |
| **PT2** | PHT1;4 | Solyc03g005530.1.1 | inorganic phosphate transporter |
| **Solyc06g048530.2.1** | GLY2 | Solyc06g048530.2.1 | hydroxyacylglutathione hydrolase cytoplasmic |
| **Solyc02g072060.1.1** | HSFA2 | Solyc02g072060.1.1 | heat stress transcription factor A-7a-like |
| **Solyc12g011320.1.1** | GSTU25 | Solyc12g011320.1.1 | glutathione S-transferase U25-like |
| **Solyc03g116120.1.1** | GSTU9 | Solyc03g116120.1.1 | glutathione S-transferase U10 |
| **Solyc12g044520.1.1** | GSTL1 | Solyc12g044520.1.1 | glutathione S-transferase L1-like isoform X1 |
| **Solyc09g072720.1.1** | AT1G10385 | Solyc09g072720.1.1 | exocyst complex component EXO84B-like |
| **Solyc02g088030.1.1** | AT1G26800 | Solyc02g088030.1.1 | E3 ubiquitin-protein ligase MPSR1-like |
| **Solyc06g010100.1.1** | SIZ1 | Solyc06g010100.1.1 | E3 SUMO-protein ligase SIZ1-like |
| **Solyc03g111280.1.1** | CYP94B3 | Solyc03g111280.1.1 | cytochrome P450 94B3-like |
| **Solyc11g065770.1.1** | CYP94B1 | Solyc11g065770.1.1 | cytochrome P450 94A2-like |
| **Solyc07g042220.1.1** | BGAL7 | Solyc07g042220.1.1 | beta-galactosidase 15-like |
| **PIN8** | PIN8 | Solyc02g087660.2.1 | auxin efflux carrier component 5 |
| **Solyc10g081890.1.1** | AT3G11680 | Solyc10g081890.1.1 | aluminum-activated malate transporter 8 |
| **Solyc11g066640.1.1** | HCT | Solyc11g066640.1.1 | agmatine coumaroyltransferase-2-like |
| **ACS8** | ACS8 | Solyc03g043890.2.1 | 1-aminocyclopropane-1-carboxylate synthase 8 |
| **Solyc06g050200.1.1** | ETG1 | Solyc06g050200.1.1 | mini-chromosome maintenance complex-binding protein |
